# Synthesis of *meso*-pyrrole-substituted corroles by condensation of 1,9-diformyldipyrromethanes with pyrrole

**DOI:** 10.3762/bjoc.18.145

**Published:** 2022-10-06

**Authors:** Baris Temelli, Pinar Kapci

**Affiliations:** 1 Hacettepe University, Department of Chemistry, Beytepe Campus, 06800, Ankara, Turkey

**Keywords:** corrole, dipyrromethane, macrocycles, metal triflates, pyrrole

## Abstract

A copper triflate-mediated approach to access copper complexes of pyrrole-substituted corroles from the reaction of 1,9-diformyldipyrromethanes and an excess amount of pyrrole is presented for the first time. This procedure is a simple and efficient way for the preparation of corroles with a polymerizable substituent on *meso*-positions.

## Introduction

Corroles, a member of contracted porphyrins, are tetrapyrrolic aromatic compounds, with the lack of one *meso*-carbon atom on the macrocycle [1–4]. This feature supplies a smaller ring cavity than in the case of porphyrins, three NH in the core, and coordination ability with high-valence transition metal ions. It is noteworthy that studies on porphyrins, which have many application areas such as photodynamic therapy and photovoltaic systems, have focused on oligomeric and polymeric structures in the last two decades [5–9]. Although such porphyrin structures have been used successfully in the development of molecular devices and functional materials, the synthesis of corrole-based analogues has been rather limited due to very few synthetic methods developed to produce corroles with polymerizable substituents at the *meso*- or beta positions [10–13].

To date, *meso*-substituted corroles have been synthesized by several methods including; (i) the condensation of pyrrole or dipyrromethanes with aldehydes [14–16], (ii) the reaction of 2,2’-bipyrrole with dipyrromethane-1,9-dicarbinols [17–18], (iii) the condensation of bipyrrole-5,5’-dicarbinols with dipyrromethanes [19], (iv) the reaction of dipyrromethane-1,9-dicarbinols with pyrrole [20–21], (v) the condensation of dipyrromethane-1-carbinols with dipyrromethanes [22] and (vi) the reaction of tripyrranes with aldehydes [23]. Although many different substituents can be attached to the *meso*-position of corroles using all these methods, to the best of our knowledge, there is no generally accepted method for the synthesis of pyrrole-substituted corroles. Very recently, we reported the synthesis of porphyrin–corrole [24–26] and porphyrin–porphyrin dyads and triads [27] using formylated porphyrin compounds. In a continuation of research activity with corroles, here we describe the first synthesis of copper complexes of *trans*-A_2_B-corroles possessing pyrrol-2-yl substituents at positions 5 and 15 by the condensation reaction of 1,9-diformylated dipyrromethanes with pyrrole in the presence of copper triflate.

## Results and Discussion

**Scheme 1:**
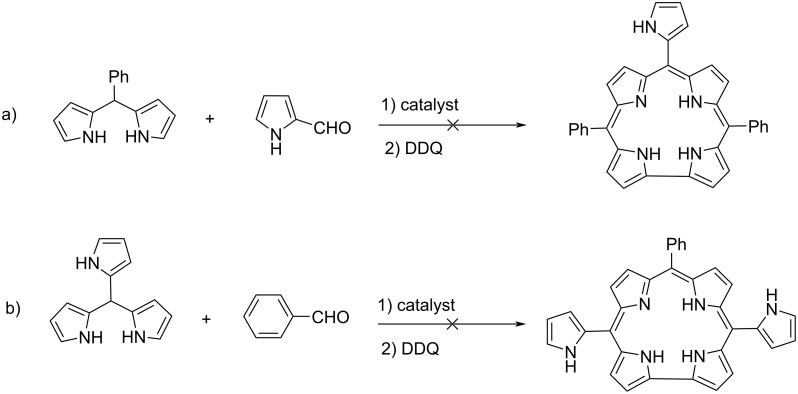
Synthetic studies to obtain mono- and dipyrrole-substituted compounds.

At the beginning of our studies, we investigated the synthesis of mono- and dipyrrole-substituted corroles via the condensation reaction of pyrrole-2-carboxaldehyde with 5-phenyldipyrromethane (Scheme 1a) and the reaction between tris(2-pyrrolyl)methane with benzaldehyde (Scheme 1b). Although we tried many reaction conditions and catalysts, unidentified product mixtures were obtained instead of corrole products in both reactions.

**Scheme 2:**
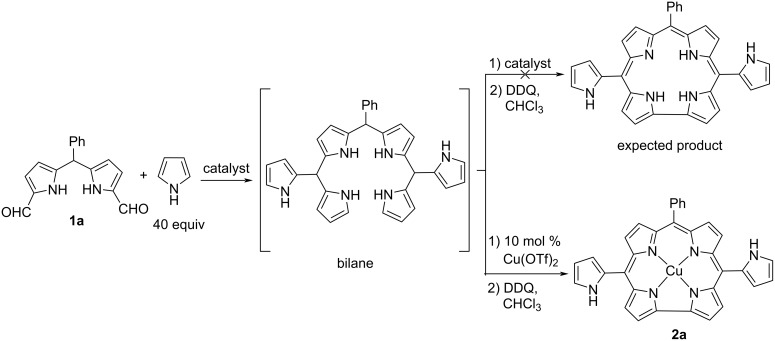
The reaction of 5-phenyl-1,9-diformyldipyrromethane (**1a**) with pyrrole.

Then, the reaction of 1,9-diformyl-5-phenyldipyrromethane (**1a**) in an excess amount of pyrrole was tested to obtain pyrrole-substituted metal-free corrole through the oxidation of the bilane intermediate by using DDQ (Scheme 2). Pyrrole was used as both reagent and solvent in these reactions. The desired product was not observed in the reaction medium when various catalysts (TFA, I_2_, AlCl_3_, InCl_3_, FeCl_3_, H_2_SO_4_, *p*-TsOH, Mont. KSF, Mont. K-10, and AgOTf) were used at different temperatures (Supporting Information File 1, Table S1). However, the copper complex of the desired product **2a** was obtained in 5% yield in the presence of Cu(OTf)_2_ catalyst at room temperature.

When the synthetic methods in the literature are examined to obtain corrole compounds, it is observed that temperature, pyrrole ratio, reaction time, catalyst type, and oxidant are important parameters on the yields of the reactions [28–32]. For this reason, optimization studies were carried out on these parameters. Based on the results of the preliminary studies, optimization studies were carried out in the presence of 10 mol % Cu(OTf)_2_ catalyst, and the effect of temperature on the synthesis of pyrrole-substituted trans-A_2_B corrole compounds was investigated in 40 equivalents of pyrrole using a reaction time of 2 hours (Table 1, entries 1–4). No product was formed as a result of increasing the reaction temperature to 40 °C. It was observed that the yield increased gradually when the reaction temperature was decreased. The yield of the product, which was obtained with 5% efficiency at room conditions and 6% at 0 °C, increased to 9% by reducing the temperature to −20 °C. The pyrrole/**1a** ratio played little role in improving the yield of the product. The yield of **2a** decreased to 4% yield when the reaction was carried out in 20 equivalents of pyrrole (Table 1, entry 5). Increasing the amount of pyrrole above 40 equivalents did not affect the reaction yield (Table 1, entries 6 and 7). Then, the effect of the reaction time before the oxidant addition on the yield of product was investigated at −20 °C in 40 equivalents of pyrrole. When the reaction time was 1 hour, the yield decreased to 4% (Table 1, entry 8). If the reaction time exceeded 2 hours, unexpectedly no desired product was found at all (Table 1, entries 9 and 10). This situation can be explained by the instability of the bilane intermediate formed in the reaction medium and its decomposition during long reaction times. It was also investigated whether the product yield would increase with the amount of catalyst since only the copper complex of the expected product could be isolated at the end of the reaction. The reaction was repeated using 20 mol %, 50 mol %, and equimolar amounts of copper triflate under previously optimized conditions. In the case of using 20 mol % copper triflate, the reaction efficiency increased to 12%, while a further increase in the amount of catalyst did not affect the yield (Table 1, entries 11–13). In order to determine the effect of the oxidant type and the oxidant amount, reactions were carried out with 3 and 4 equivalents of DDQ and *p*-chloranil. While more than 2 equivalents of DDQ did not have a positive effect on the reaction yield (Table 1, entries 14 and 15), *p*-chloranil formed a product with a lower yield than DDQ (Table 1, entries 16–18). The activities of different copper catalysts were also tested in the model reaction. Only CuCl_2_ formed the product in 5% yield and the other salts did not catalyze the reaction (Table 1, entries 19–22).

**Table 1:** Optimization of reaction conditions.^a^

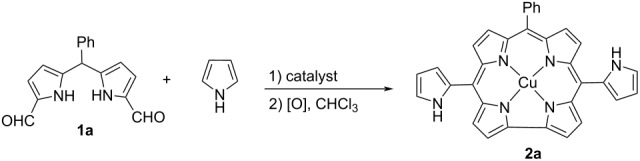							

Entry	Catalyst	Catalyst amount (%)	Temp (°C)	Pyrrole/**1a**	Time (h)	Oxidant (oxidant/**1a**)	Yield (%)^b^

1	Cu(OTf)_2_	10	40	40	2	DDQ (2)	–
2	Cu(OTf)_2_	10	rt	40	2	DDQ (2)	5
3	Cu(OTf)_2_	10	0	40	2	DDQ (2)	6
4	Cu(OTf)_2_	10	−20	40	2	DDQ (2)	9
5	Cu(OTf)_2_	10	−20	20	2	DDQ (2)	4
6	Cu(OTf)_2_	10	−20	60	2	DDQ (2)	9
7	Cu(OTf)_2_	10	−20	80	2	DDQ (2)	9
8	Cu(OTf)_2_	10	−20	40	1	DDQ (2)	4
9	Cu(OTf)_2_	10	−20	40	4	DDQ (2)	–
10	Cu(OTf)_2_	10	−20	40	6	DDQ (2)	–
11	Cu(OTf)_2_	20	−20	40	2	DDQ (2)	12
12	Cu(OTf)_2_	50	−20	40	2	DDQ (2)	12
13	Cu(OTf)_2_	100	−20	40	2	DDQ (2)	12
14	Cu(OTf)_2_	20	−20	40	2	DDQ (3)	12
15	Cu(OTf)_2_	20	−20	40	2	DDQ (4)	12
16	Cu(OTf)_2_	20	−20	40	2	*p*-chloranil (2)	10
17	Cu(OTf)_2_	20	−20	40	2	*p*-chloranil (3)	10
18	Cu(OTf)_2_	20	−20	40	2	*p*-chloranil (4)	10
19	CuCl_2_	100	−20	40	2	DDQ (2)	5
20	CuCl	100	−20	40	2	DDQ (2)	–
21	Cu(OAc)_2_	100	−20	40	2	DDQ (2)	–
22	Cu(NO_3_)_2_	100	−20	40	2	DDQ (2)	–

^a^Reaction conditions: **1a** (0.36 mmol, 0.10 g), pyrrole (14.4 mmol, 0.97 g, 1 mL), CHCl_3_ (2 mL). ^b^Isolated yield.

With the best conditions in our hands (Table 1, entry 11), different diformylated dipyrromethanes were subjected to condensation reactions. Electron-withdrawing 4-chlorophenyl, pentafluorophenyl, and 4-nitrophenyl-substituted corrole compounds were isolated in 13% yields (Table 2, entries 2–4). While electron-donating 4-methoxyphenyl (**2e**) and *p*-tolyl-substituted corrole (**2f**) were isolated in 8% yield and 12% yields respectively, a *p*-bromophenyl substituent resulted in a mixture of undefined products after the reaction. This might be due to scrambling, which is an acid-catalyzed rearrangement of the substituent in intermediates of the condensation reaction.

**Table 2:** Synthesis of pyrrole-substituted corroles.^a^

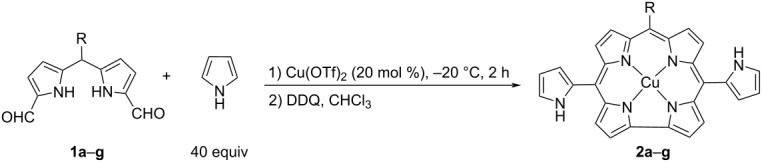			

Entry	R	**2**	Yield (%)^b^

1	C_6_H_5_	**2a**	12
2	4-ClC_6_H_4_	**2b**	13
3	C_6_F_5_	**2c**	13
4	4-NO_2_C_6_H_4_	**2d**	13
5	4-CH_3_OC_6_H_4_	**2e**	8
6	4-CH_3_C_6_H_4_	**2f**	12
7	4-BrC_6_H_4_	**2g**	–

^a^Reaction conditions: **1a**–**g** (0.36 mmol), pyrrole (14.4 mmol, 0.97 g, 1 mL), Cu(OTf)_2_ (0.072 mmol, 0.026 g), DDQ (0.72 mmol, 0.16 g ), CHCl_3_ (2 mL). ^b^Isolated yield.

**Figure 1:**
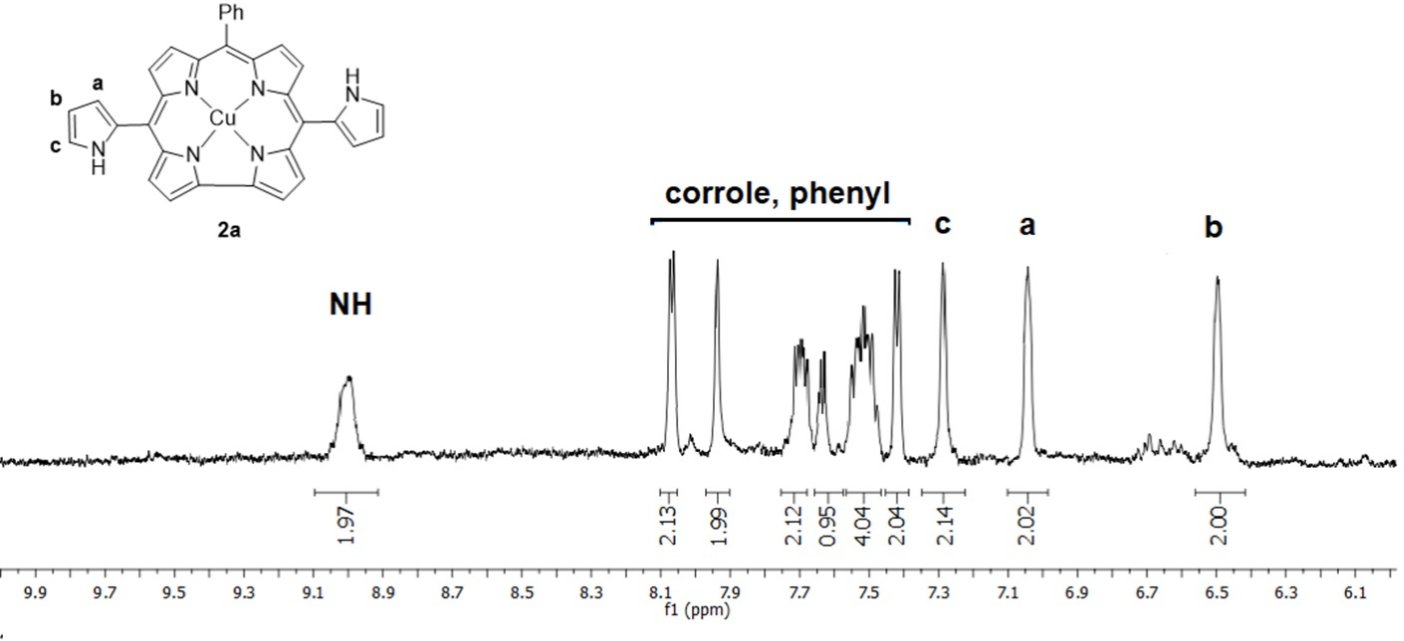
^1^H NMR spectrum of **2a** in THF-*d*_8_.

The structures of the *meso*-pyrrole substituted corroles were identified by using ^1^H NMR, ^1^H,^1^H-COSY NMR and HRMS techniques (see Supporting Information File 1). The ^1^H NMR spectrum of **2a** is shown in Figure 1. As expected, pyrrole C4, C3, C5 and NH protons appeared at 6.51, 7.05, 7.27 and 8.90 ppm, respectively. Coupling of all pyrrole protons can be seen in the ^1^H,^1^H-COSY NMR spectrum (Supporting Information File 1, Figure S3). The β-protons of the corrole macrocycle and the phenyl group gave signals between 7.40–8.20 ppm.

**Figure 2:**
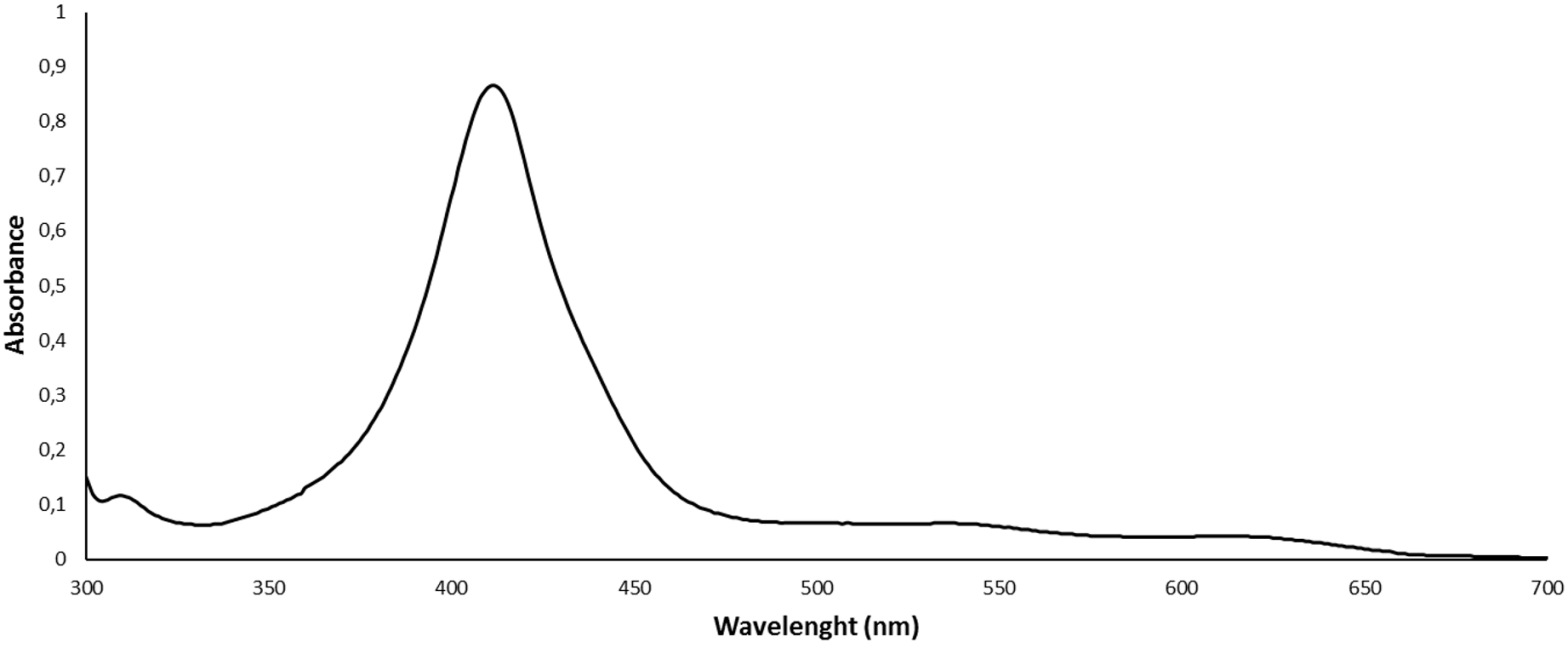
Electronic absorption spectrum of **2a** in CHCl_3_.

Electronic absorption spectra of corroles **2a**–**g** were recorded in CHCl_3_ at 2.0 × 10^−5^ M. The Soret bands of all compounds are located between 410–420 nm (see Supporting Information File 1). The Q bands of the compounds are seen as broad absorptions in the 500–700 nm region. Figure 2 shows the absorption spectrum of **2a** with a strong Soret band at 412 nm and weak Q-bands at 542 and 611 nm.

**Scheme 3:**
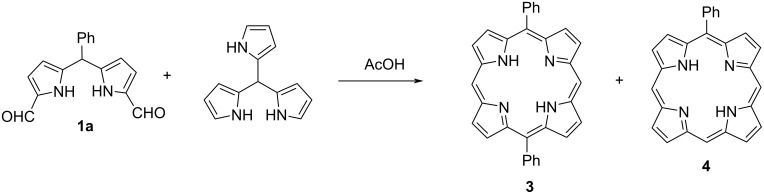
[2 + 2] Mac Donald type condensation reaction.

After the synthesis of corrole compounds, we tried to extent our studies to obtain *meso*-pyrrole-substituted porphyrin compounds. For this purpose, the MacDonald [2 + 2] porphyrin macrocyclization reaction of 1,9-diformyl-5-phenyl dipyrromethane (**1a**) with tris(2-pyrrolyl)methane was investigated by changing the reaction conditions in the presence of various acids such as acetic acid, hydrochloric acid and *p*-toluenesulfonic acid (Scheme 3). Among these reactions, the reaction in acetic acid resulted in 5,15-diphenylporphyrin (**3**) and 5-phenylporphyrin (**4**) in 4% and 1% yields, respectively. No pyrrole-substituted porphyrin product was detected. The structures of compounds **3** [33] and **4** [34] are in agreement with the literature data.

## Conclusion

In summary, we report the first example of copper complexes of A_2_B-type pyrrole substituted corroles. We believe that the placement of the polymerizable pyrrole as a conjugated substituent to the macrocycle is an important contribution to the polymerization of corroles and the expansion of the usage areas of these compounds. Further studies on the production of mono pyrrole metal-free corrole compounds and the polymerization reactions of the obtained compounds are ongoing in our laboratory.

## Experimental

### General information

All reactions were performed under N_2_ atmosphere. All reagents and solvents were of reagent grade. The NMR spectra were recorded in CDCl_3_ and THF-*d*_8_ on a Bruker AV Ultra Shield 400 MHz instrument. Absorption spectra were obtained with PG T80. NMR data are represented as follows: chemical shift (ppm), multiplicity (s = singlet, brs = broad singlet, d = doublet, t = triplet, q = quartet, m = multiplet), coupling constants in hertz (Hz). IR spectra were recorded on FTIR spectrometer (Thermo Scientific, Nicolet IS10). HRMS were measured in ESI mode and the mass analyzer of the HRMS was TOF (Agilent 6224 TOF LC–MS). Flash column chromatography was performed on silica gel (230–400 mesh). 5-Substituted dipyrromethanes [35] and 1,9-diformyldipyrromethanes [36] were prepared according to literature methods and their spectral data matched literature values.

### General synthetic procedure for pyrrole-substituted corroles

A solution of 1,9-diformyldipyrromethanes **1a**–**g** (0.36 mmol) and pyrrole (14.4 mmol, 0.97 g, 1 mL) was cooled under N_2_ at −20 °C for 1 h. Cu(OTf)_2_ (0,072 mmol, 0.026 g) was added to the mixture at the same temperature and stirred for 2 h. A solution of DDQ (0.72 mmol, 0.16 g) in 2 mL CHCl_3_ was added to the reaction. The reaction was removed from the cold bath and left to stir overnight. The mixture passed from the silica column to remove Cu(OTf)_2_. The solvent was removed under reduced pressure and the crude product was purified by flash column chromatography over silica gel with CH_2_Cl_2_/hexane (1:1).

### Synthesis of **3** and **4** by [2 + 2] MacDonald coupling reaction

A solution of 5-phenyl-1,9-diformyldipyrromethane (**1a**, 0.40 mmol, 0.11 g) and tris(2-pyrrolyl)methane (0.40 mmol, 0.085 g) in 60 mL HOAc was stirred for 2 hours in the dark and 4 g NaOAc was added. The reaction was stirred for 12 h. in the dark. Solvent was removed by distillation in vacuum and 120 mL MeOH and 2 mL concd H_2_SO_4_ was added to the residue. The mixture was refluxed for 12 h. The solvent was removed under reduced pressure and the crude product was purified by flash column chromatography over silica gel with CHCl_3_/hexane (1:1).

## Supporting Information

File 1 Table S1 and experimental part.
