# Supplementary material for: Synthesis of meso-pyrrole-substituted corroles by condensation of 1,9-diformyldipyrromethanes with pyrrole
Source: Beilstein J Org Chem. 2022 Oct 6;18:1403–9. doi: 10.3762/bjoc.18.145 (PMC9551205; doi:10.3762/bjoc.18.145)
Supplement: File 1 — Table S1 and experimental part. [file Beilstein_J_Org_Chem-18-1403-s001.pdf]

BEILSTEIN JOURNAL OF ORGANIC CHEMISTRY

Supporting Information

for

Synthesis of *meso*-pyrrole-substituted corroles by condensation of 1,9-diformyldipyrromethanes with pyrrole

Baris Temelli and Pinar Kapci

Beilstein J. Org. Chem. **2022,** *18,* 1403–1409. doi:10.3762/bjoc.18.145

Table S1 and experimental part

License and Terms: This is a supporting information file under the terms of the Creative Commons Attribution License (https://creativecommons.org/licenses/by/4.0). Please note that the reuse, redistribution and reproduction in particular requires that the author(s) and source are credited and that individual graphics may be subject to special legal provisions.
The license is subject to the *Beilstein Journal of Organic Chemistry* terms and conditions: (https://www.beilstein-journals.org/bjoc/terms)

Table S1: Unsuccessful acid catalysts for the synthesis of pyrrole-substituted corrole[a].

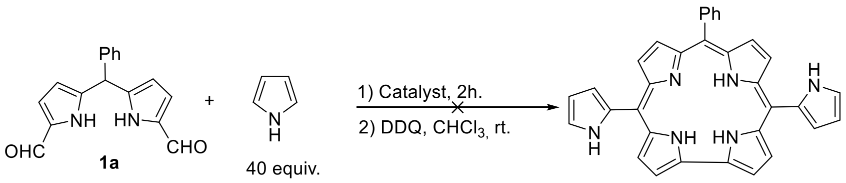

Entry	Catalyst	equiv of catalyst	Temperature (°C)
1	TFA[b]	0.1	−20
2	TFA[b]	0.1	0
4	I_2[c]	0.1	−20
5	I_2[b]	0.1	0
6	I_2[b]	0.1	rt
7	$InCl_3$[b]	0.1	−20
8	$InCl_3$[b]	0.1	0
9	$AlCl_3$[c]	1	−20
10	$AlCl_3$[b]	1	0
11	$AlCl_3$[b]	1	rt
12	$FeCl_3$[c]	1	−20
13	$FeCl_3$[b]	1	0
14	$FeCl_3$[b]	1	rt
15	H_2SO_4[b]	0.1	−20
16	H_2SO_4[b]	0.1	0
17	*p*-TsOH[b]	0.1	−20
18	*p*-TsOH[b]	0.1	0
19	Mont. K-10[b,d]		−20
20	Mont. K-10[b,d]		0
21	Mont. KSF[c,d]		−20
22	Mont. KSF[c,d]		0
23	Mont. KSF[c,d]		rt
24	Ag(OTf)[c]	0.1	−20
25	Ag(OTf)[b]	0.1	0

[a]Reaction conditions: **1a** (0.36 mmol, 0.10 g), pyrrole (14.4 mmol, 0.97, 1 mL), DDQ (0.72 mmol, 0.16 g), $CHCl_3$ (2 mL); [b]Unidentified oligomeric products were obtained. [c]No product was observed on TLC and the starting materials were recovered without the oxidation step; [d]0.36 g of clay catalyst was used.

[10-Phenyl-5,15-dipyrrolylcorrolato]copper(III) (2a): Green solid, mp> 250 ^{o}C; 24 mg (0.043 mmol), 12% yield; UV/Vis ($CHCl_3$) λ_{max} (ε x 10^{-4} L mol^{-1} cm^{-1}): 412 (4.33), 542 (0.16), 611 (0.11) nm; FTIR (ATR): 702, 748, 842, 865, 980, 1005, 1058, 1168, 1276, 1460, 1590, 2843, 2960 cm^{-1}; ^{1}H NMR (400 MHz, THF-d_8): δ 6.51 (d, ^{3}J= 1.2 Hz, 2H), 7.05 (s, 2H), 7.27 (s, 2H), 7.41 (d, ^{3}J= 4.6 Hz, 2H), 7.45-7.60 (m, 4H), 7.61-7.65 (m, 1H), 7.66-7.76 (m, 2H), 7.94 (d, ^{3}J= 1.2 Hz, 2H), 8.07 (d, ^{3}J= 4.2 Hz, 2H), 8.90 (brs, 2H, NH); HRMS (ESI) m/z calcd for $C_{33}H_{21}CuN_6$ [M] 564.1124, found 564.1072.

[10-(4-Chlorophenyl)-5,15- dipyrrolylcorrolato]copper(III) (2b): Green solid, mp> 250 ^{o}C; 28 mg (0.047 mmol), 13% yield; UV/Vis ($CHCl_3$) λ_{max} (ε x 10^{-4} L mol^{-1} cm^{-1}): 410 (4.58), 544 (0.30), 612 (0.20) nm; FTIR (ATR): 690, 850, 882, 1014, 1068, 1170, 1282, 1460, 2840, 2958 cm^{-1}; ^{1}H NMR ($CDCl_3$): δ 6.49 (d, ^{3}J= 3.0 Hz, 2H), 7.39 (d, ^{3}J= 4.3 Hz, 2H), 7.46 (d, ^{3}J= 7.6 Hz, 2H), 7.65-7.74 (m, 8H), 7.92-7.95 (m, 2H), 8.03-8.08 (m, 2H), 8.91 (brs, 2H, NH); HRMS (ESI) m/z calcd for $C_{33}H_{20}ClCuN_6$ [M] 598.0734, found 598.0752.

[10-(Pentafluorophenyl)-5,15- dipyrrolylcorrolato]copper(III) (2c): Green solid, mp> 250 ^{o}C; 26 mg (0.047 mmol), 13% yield; UV/Vis ($CHCl_3$) λ_{max} (ε x 10^{-4} L mol^{-1} cm^{-1}): 410 (4.64), 534 (0.20) nm; FTIR (ATR): 712, 812, 898, 1021, 1068, 1170, 1198, 1455, 2988, 3012 cm^{-1}; ^{1}H NMR ($CDCl_3$): δ 6.49 (brs, 2H), 7.05 (brs, 2H), 7.41 (brs, 2H), 7.54 (brs, 2H), 7.73 (brs, 2H), 7.93 (brs, 2H), 8.05 (brs 2H), 8.89 (brs, 2H); HRMS (ESI) m/z calcd for $C_{33}H_{16}CuF_5N_6$ [M] 654.0653, found 654.0593.

[10-(4-Nitrophenyl)-5,15- dipyrrolylcorrolato]copper(III) (2d): Green solid, mp> 250 °C; 29 mg (0.047 mmol), 13% yield; UV/Vis ($CHCl_3$) λ_{max} (ε x 10^{-4} L mol^{-1} cm^{-1}): 420 (3.88), 551 (0.15) nm; FTIR (ATR): 734, 823, 941, 1013, 1070, 1187, 1381, 1545, 2988, 2990 cm^{-1}; ^1H NMR ($CDCl_3$): δ 6.49 (s, 2H), 6.96 (s, 2H), 7.65 (s, 2H), 7.76 (s, 2H), 8.02 (s, 2H), 8.14 (s, 2H), 8.19 (s, 2H), 8.43-8.50 (m, 4H), 8.84 (brs, 2H, NH); HRMS (ESI) m/z calcd for $C_{33}H_{20}CuN_7O_2$ [M] 609.0974, found 609.0923.

[10-(4-Metoxyphenyl)-5,15- dipyrrolylcorrolato]copper(III) (2e): Green solid, mp> 250 °C; 17 mg (0.029 mmol), 8% yield; UV/Vis ($CHCl_3$) λ_{max} (ε x 10^{-4} L mol^{-1} cm^{-1}): 414 (4.27), 536 (0.17), 608 (0.11) nm; FTIR (ATR): 760, 845, 935, 1025, 1077, 1214, 1490, 1575, 2820, 2992 cm^{-1}; ^1H NMR ($CDCl_3$): δ 4.09 (s, 3H, OCH_3), 6.49 (s, 2H), 7.41 (s, 2H), 7.43-7.55 (m, 10H), 7.93 (s, 2H), 8.04 (s, 2H), 8.90 (brs, 2H, NH); HRMS (ESI) m/z calcd for $C_{34}H_{23}CuN_6O$ [M] 594.1229, found 594.1276.

[10-(Tolyl)-5,15- dipyrrolylcorrolato]copper(III) (2f): Green solid, mp> 250 °C; 25 mg, 12% yield (0.043 mmol); UV/Vis ($CHCl_3$) λ_{max} (ε x 10^{-4} L mol^{-1} cm^{-1}): 412 (4.09), 534 (0.28), 614 (0.18) nm; FTIR (ATR): 712, 750, 815, 843, 981, 1005, 1060, 1163, 1276, 1454, 1583, 2855, 2981 cm^{-1}; ^1H NMR ($CDCl_3$): δ 2.94 (s, 3H, CH_3), 6.49 (d, ^3J= 2.5 Hz, 2H), 7.35-7.40 (m, 3H), 7.43-7.48 (m, 2H), 7.52 (s, 3H), 7.63-7.68 (m, 2H), 7.69-7.73 (m, 2H), 7.90-7.94 (m, 2H), 8.05 (d, $^3J= 4.2$ Hz, 2H), 8.89 (brs, 2H, NH); HRMS (ESI) m/z calcd for $C_{34}H_{23}CuN_6$ [M] değeri 578.1280, found 578.1225.

5,15-Diphenylporphyrin (**3**): Purple solid, mp> 250 °C; 8 mg (0.017 mmol), 4% yield; ^1H NMR (400 MHz,$CDCl_3$): δ -3.18 (s, 2H, NH), 7.74-7.75 (m, 6H), 8.20-8.22 (m, 4H), 9.02 (d, J= 4.5 Hz, 4H, β-H), 9.34 (d, J= 4.5 Hz, 4H, β-H), 10.26 (s, 2H, meso-H). Analytical data were as described in the literature [1].

5-Phenylporphyrin (**4**): Purple solid, mp> 250 °C; 2 mg (0.005 mmol), 1% yield; ^1H NMR (400 MHz,$CDCl_3$): δ -3.48 (s, 2H, NH), 7.75-7.85 (m, 3H), 8.25-8.30 (m, 2H), 9.10-9.20 (m, 2H), 9.40-9.50 (m, 2H), 9.52-9.60 (m, 4H), 10.27 (s, 1H), 10.37 (s, 2H);); HRMS (ESI) m/z calcd for $C_{26}H_{18}N_4$ $[M+H]^+$ 387.1604, found 387.1632. Analytical data were as described in the literature. [2]

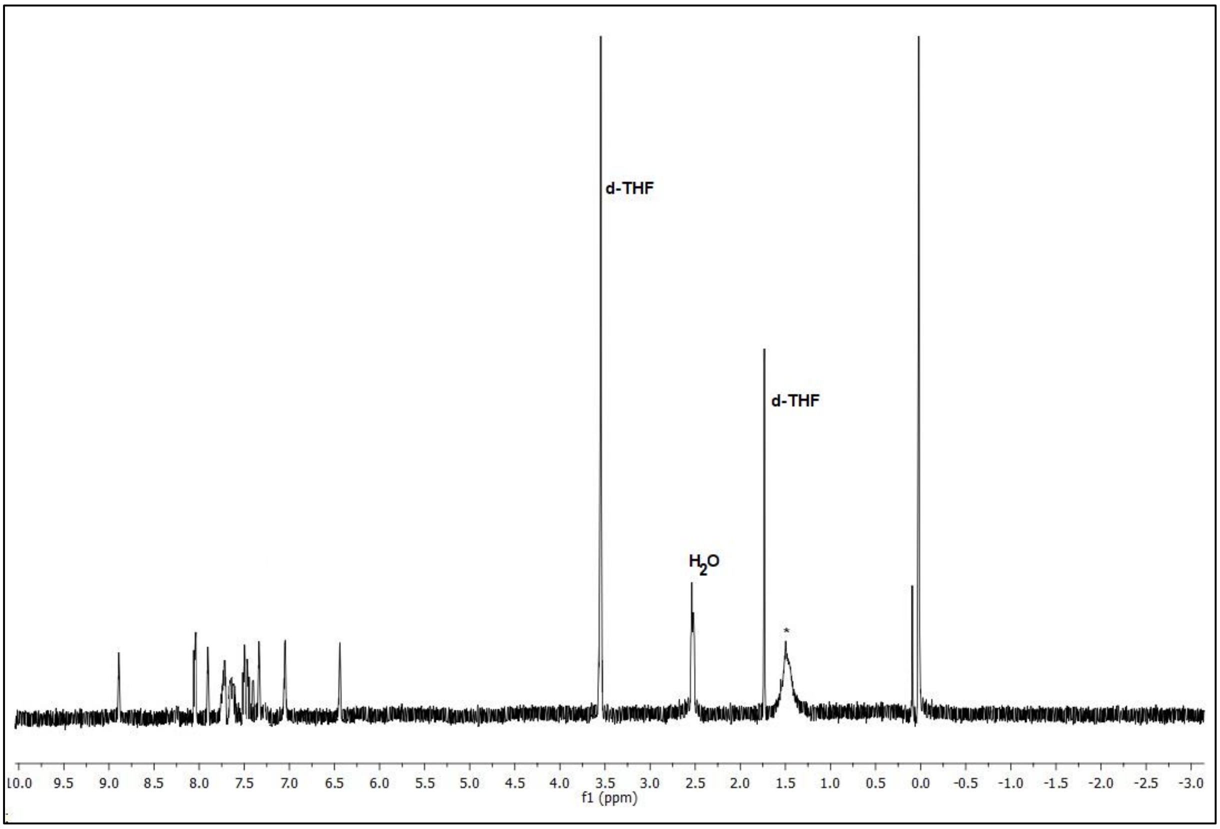

Figure S1:^{1}H NMR spectrum of **2a** (−3–10 ppm, Solvent: THF-d_8).

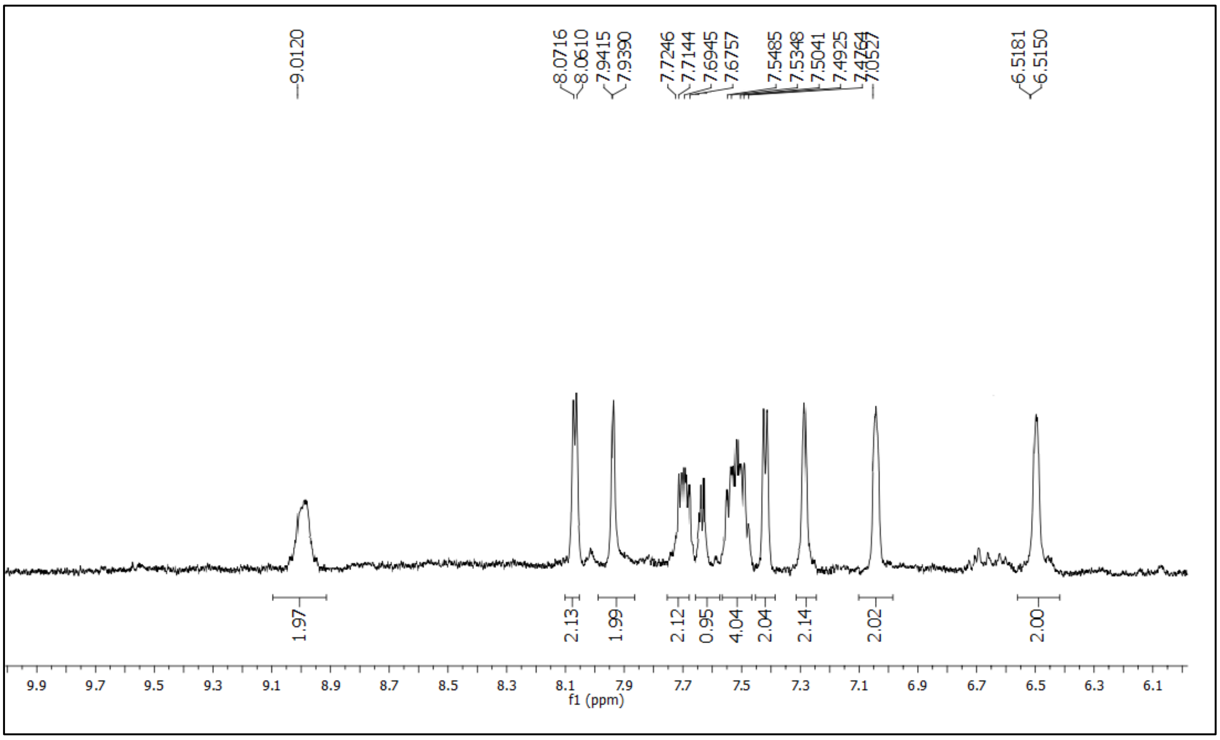

Figure S2: ^{1}H NMR spectrum of **2a** (6.0–10.0 ppm, Solvent: THF-d_8).

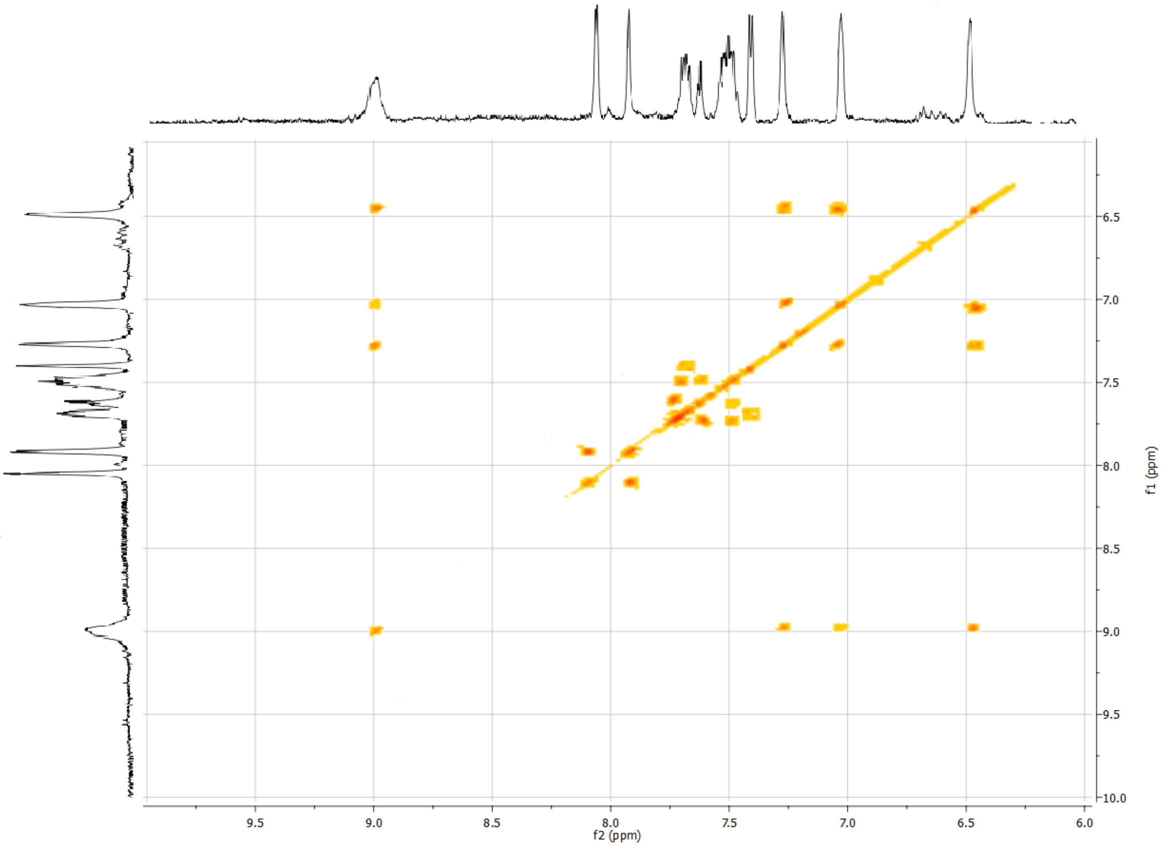

Figure S3:^{1}H-^{1}H COSY NMR spectrum of **2a** (6.0–10.0 ppm, Solvent: THF-d_8).

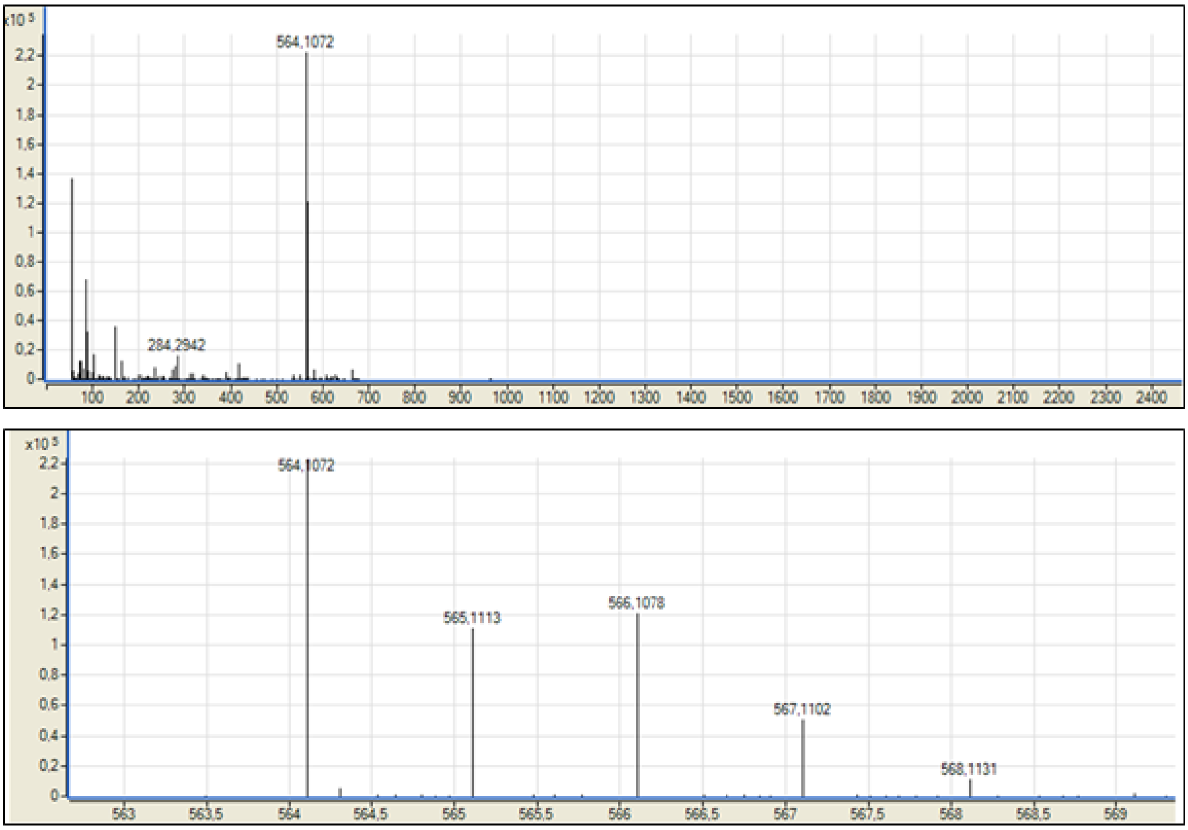

Figure S4: ESI HRMS (positive mode) of compound **2a**.

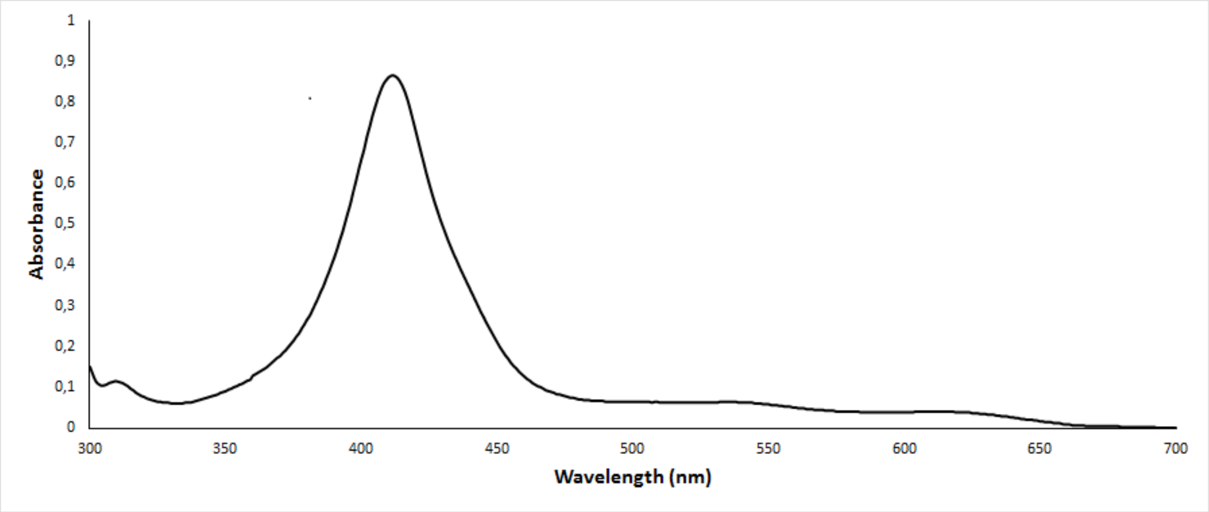

Figure S5: Electronic absorption spectra of **2a** in $CHCl_3$.

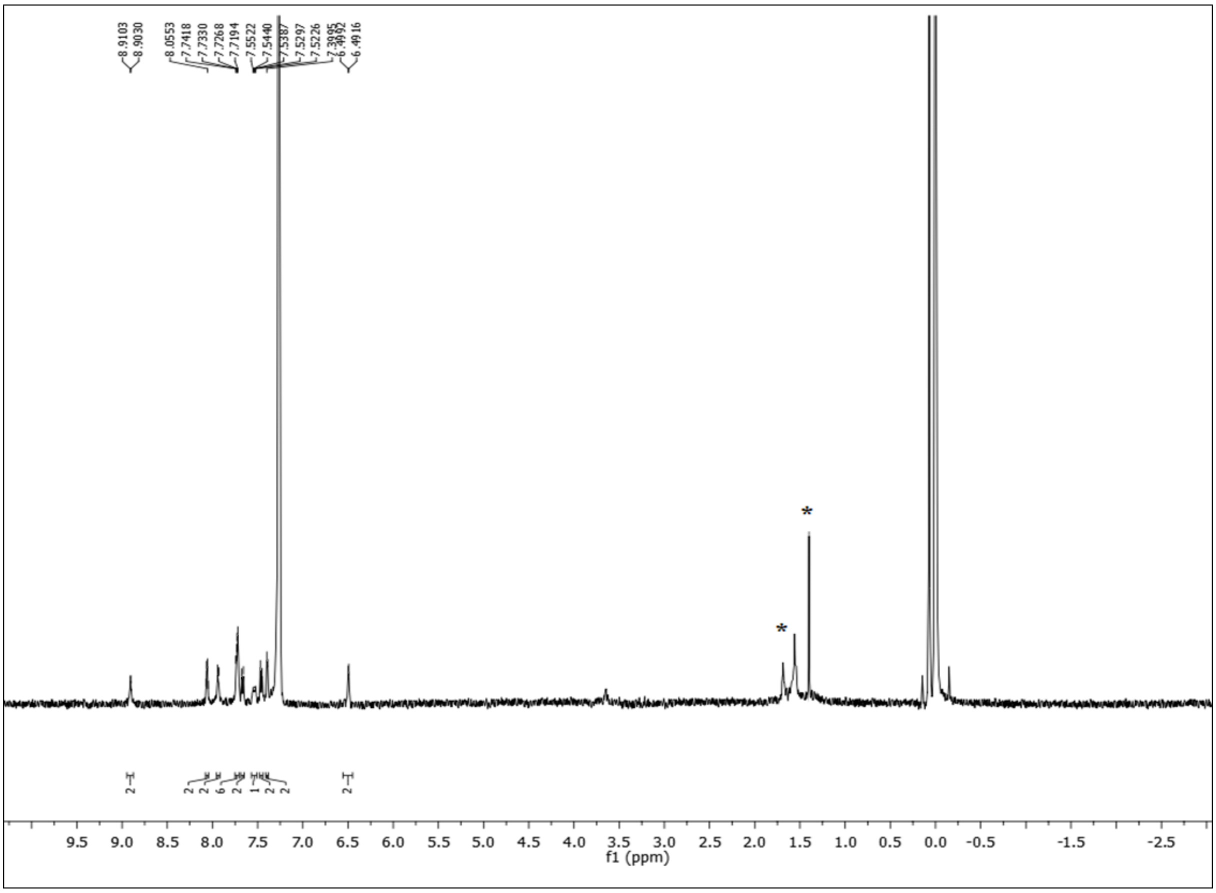

Figure S6:^{1}H NMR spectrum of **2b** (−3–10 ppm, Solvent: $CDCl_3$).

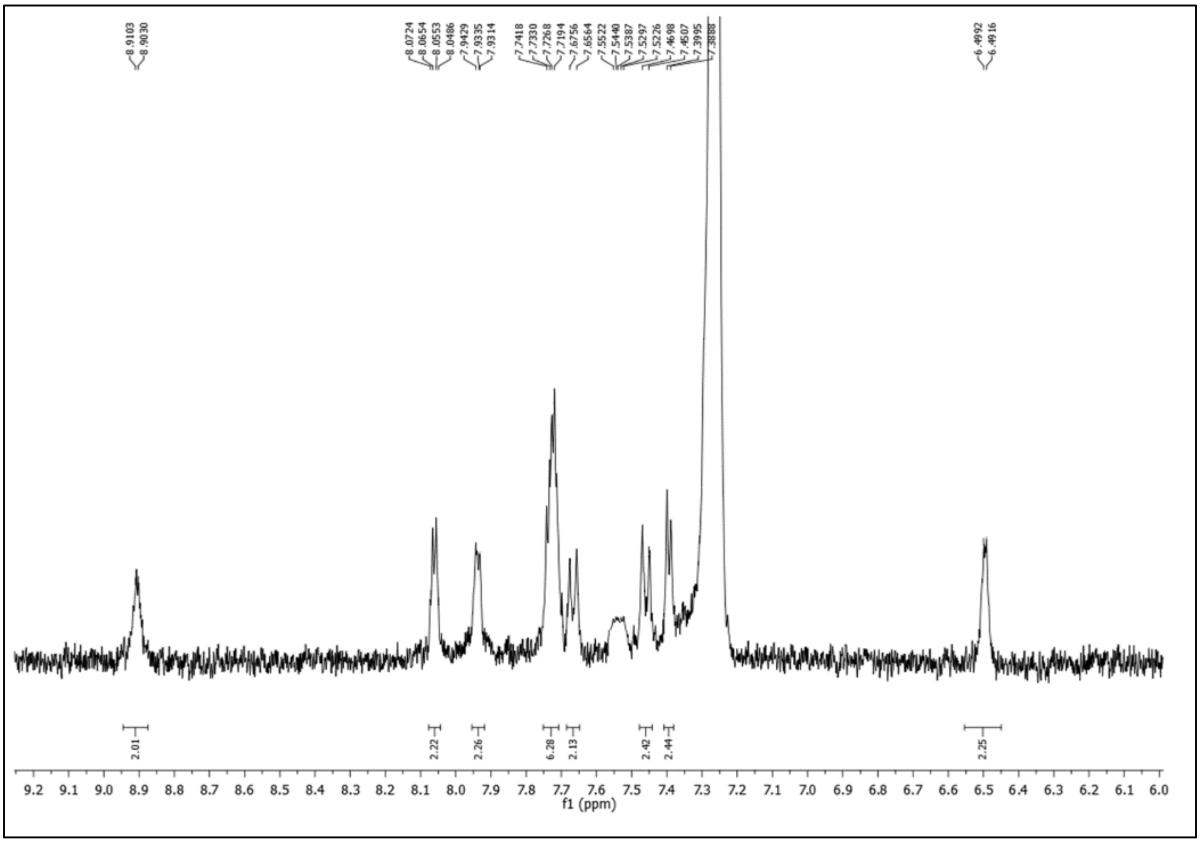

Figure S7: ^{1}H NMR spectrum of **2b** (6.0–9.3 ppm, Solvent: $CDCl_3$).

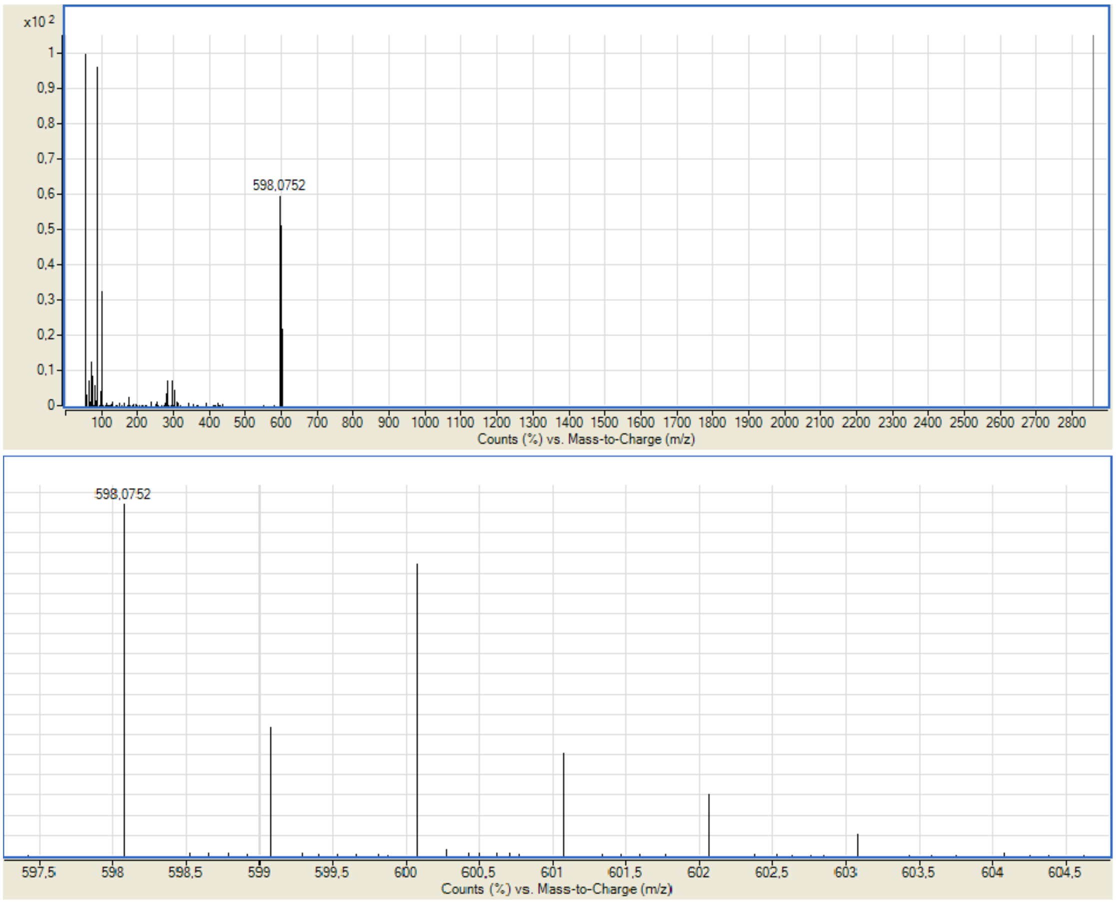

Figure S8: ESI HRMS (positive mode) of compound **2b**.

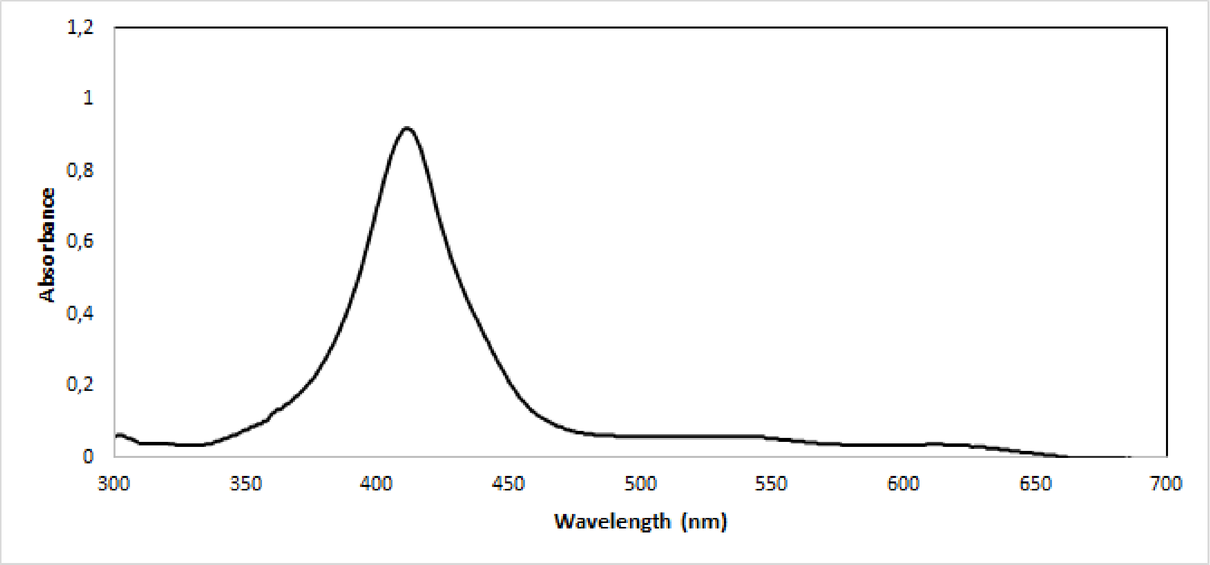

Figure S9: Electronic absorption spectra of **2b** in $CHCl_3$.

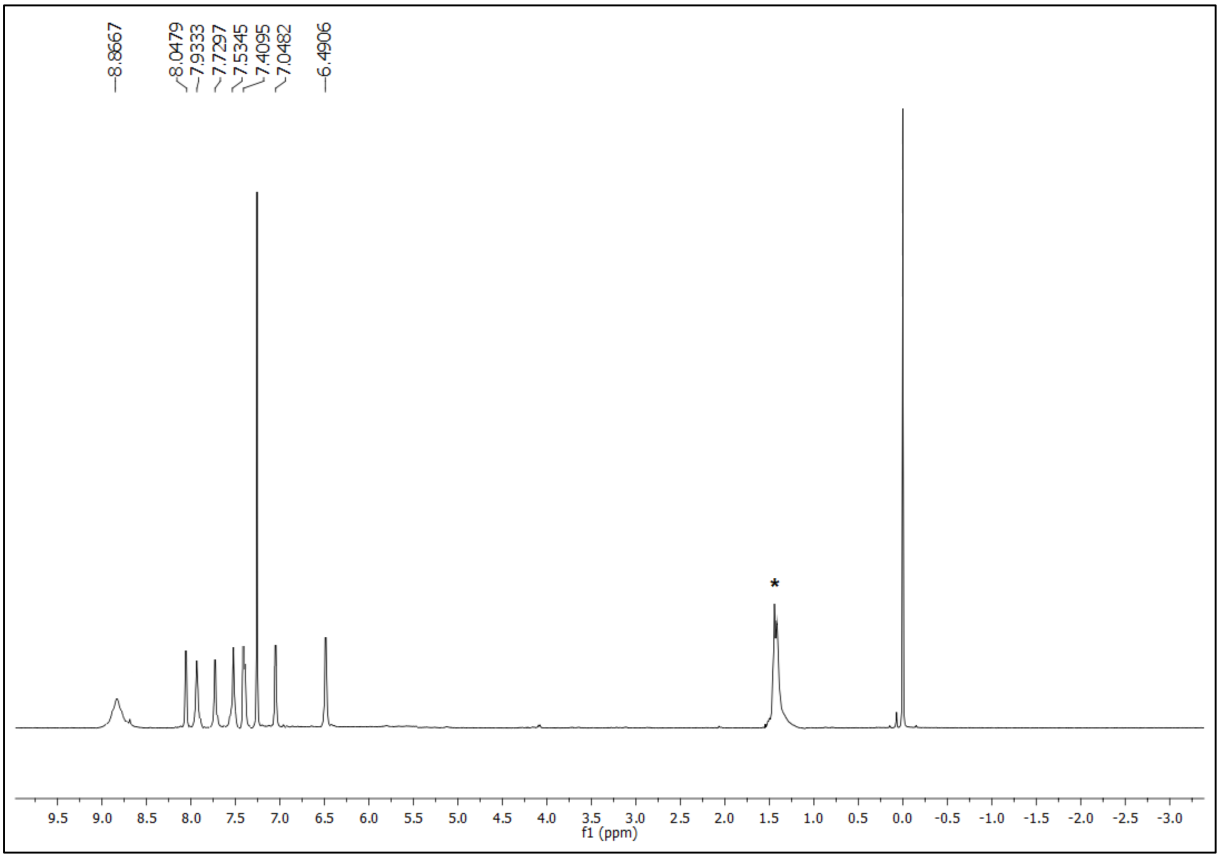

Figure S10: ^{1}H NMR spectrum of **2c** (−3–10 ppm, Solvent: $CDCl_3$).

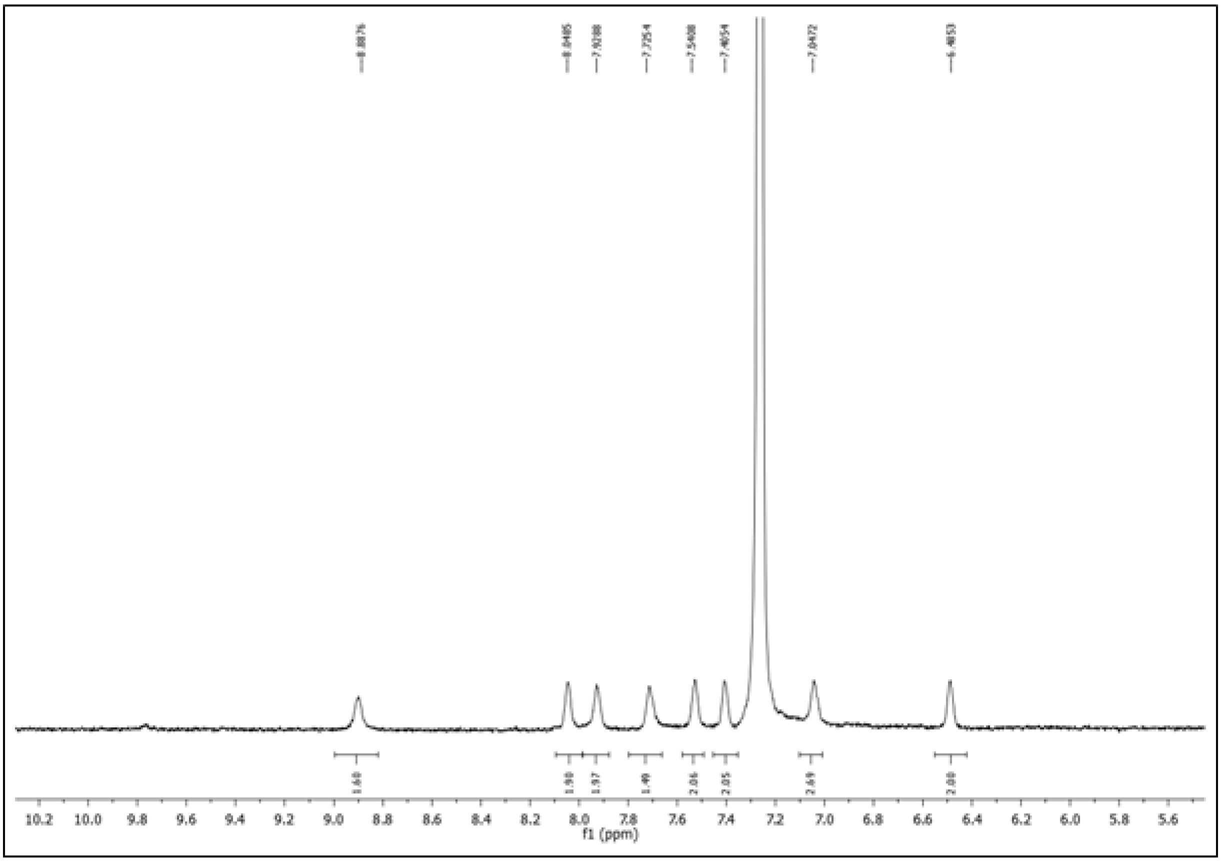

Figure S11: ^{1}H NMR spectrum of **2c** (5.5–10.2 ppm, Solvent: $CDCl_3$).

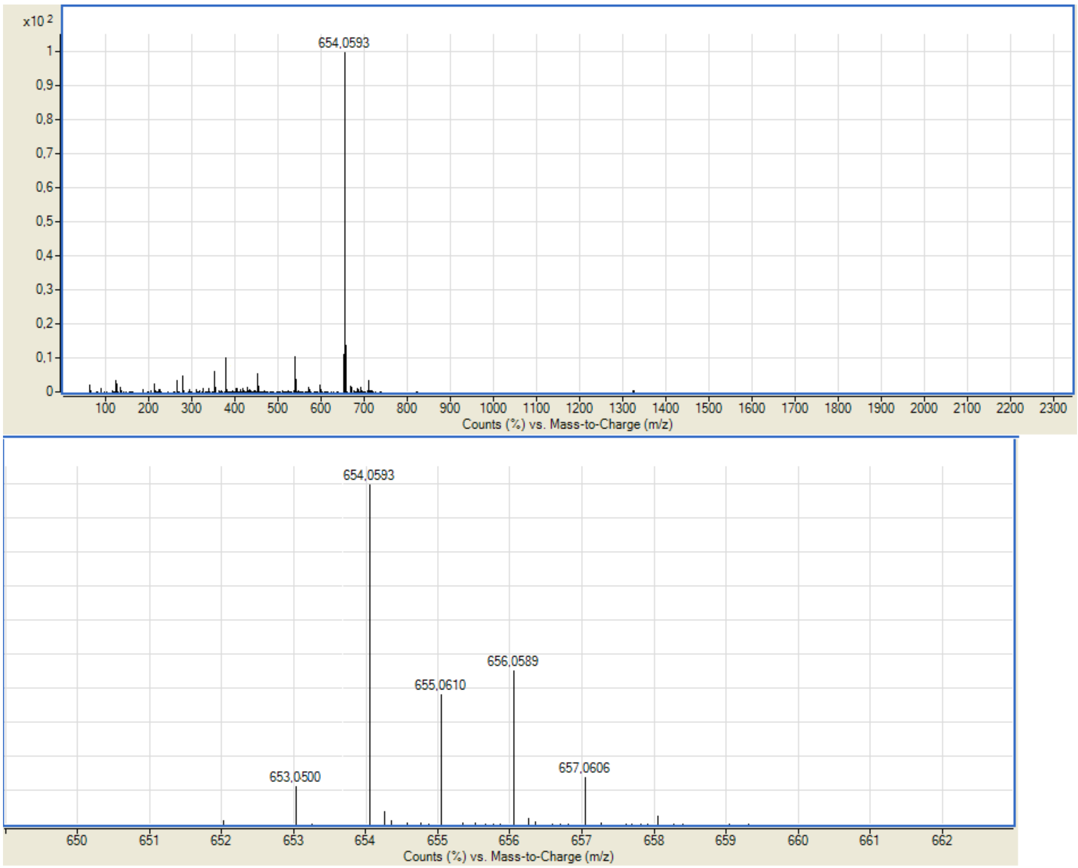

Figure S12: ESI HRMS (positive mode) of compound **2c**.

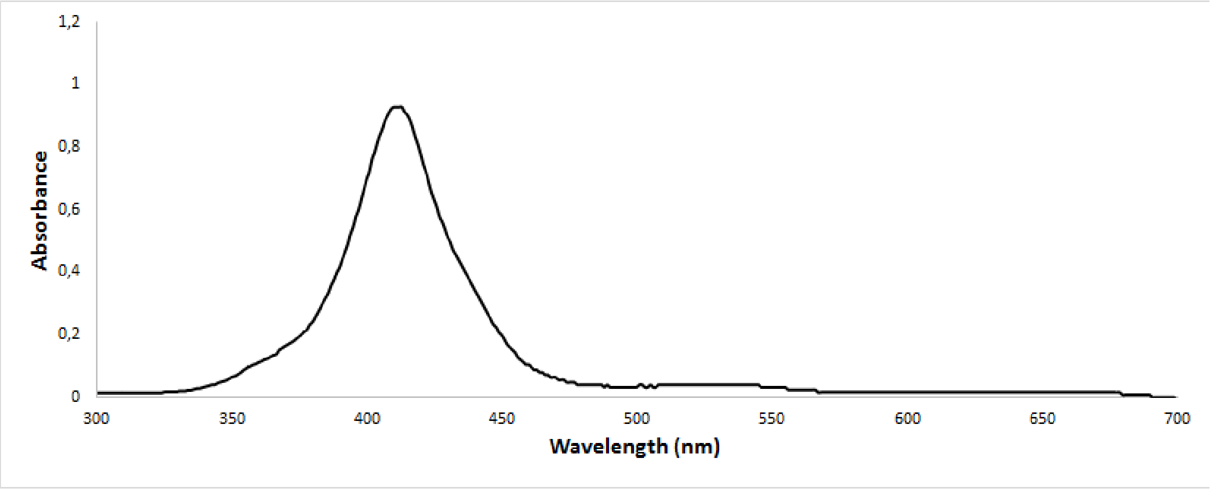

Figure S13: Electronic absorption spectra of **2c** in $CHCl_3$.

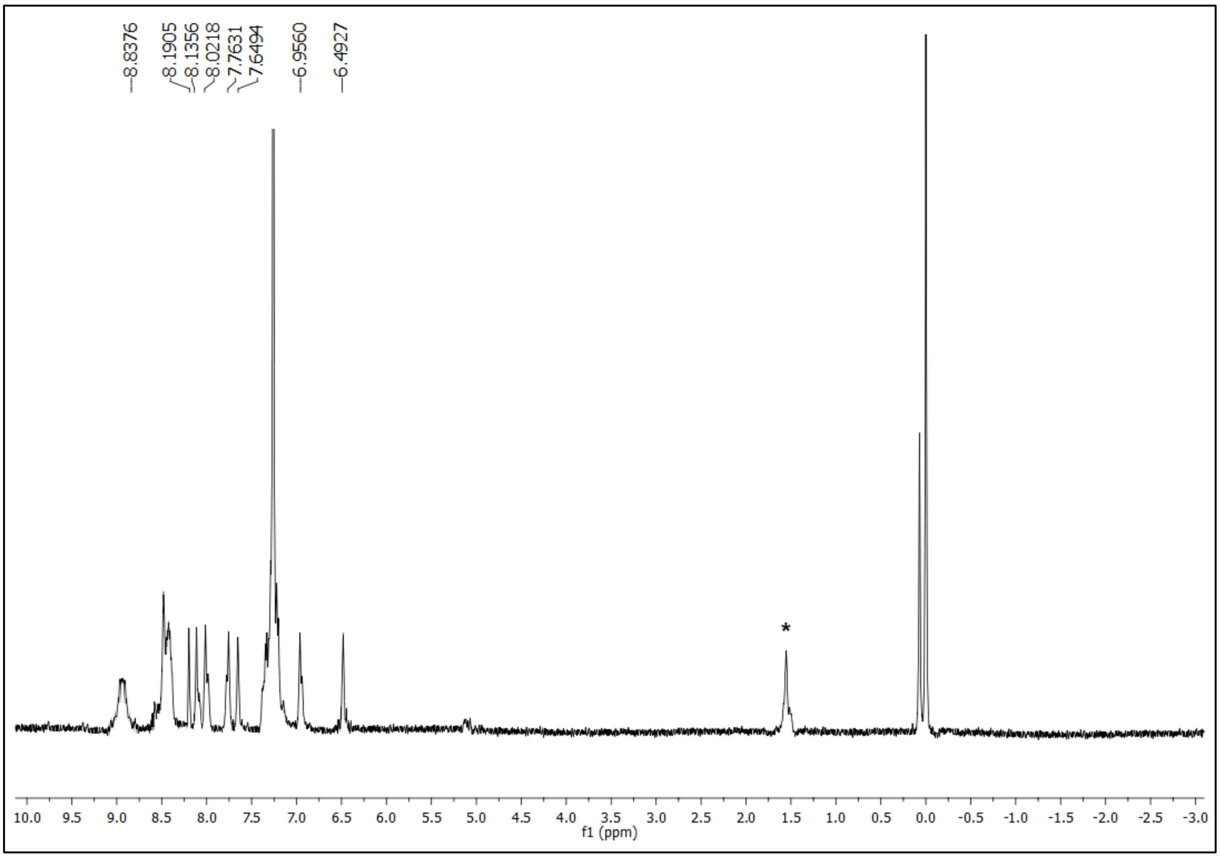

Figure S14: ^{1}H NMR spectrum of **2d** (−3–10 ppm, Solvent: $CDCl_3$).

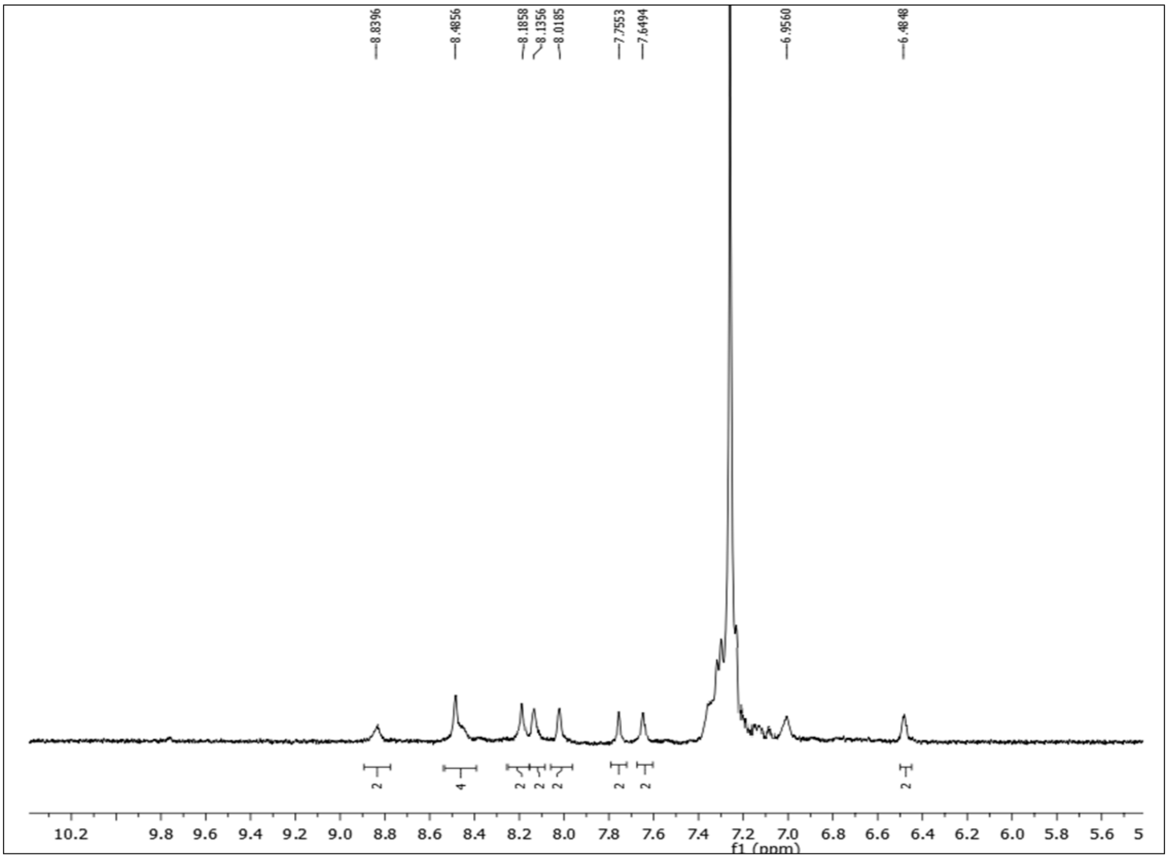

Figure S15: ^{1}H NMR spectrum of **2d** (6.0–9.3 ppm, Solvent: $CDCl_3$).

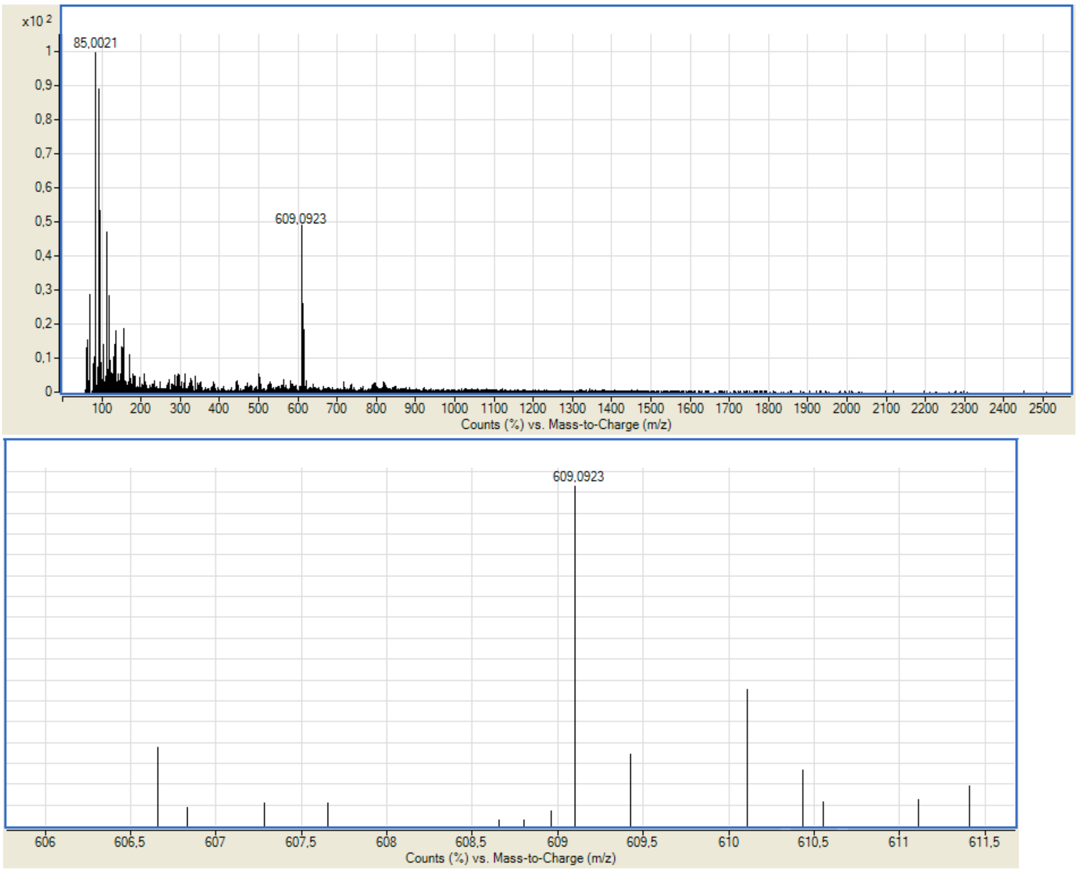

Figure S16: ESI HRMS (positive mode) of compound **2d**.

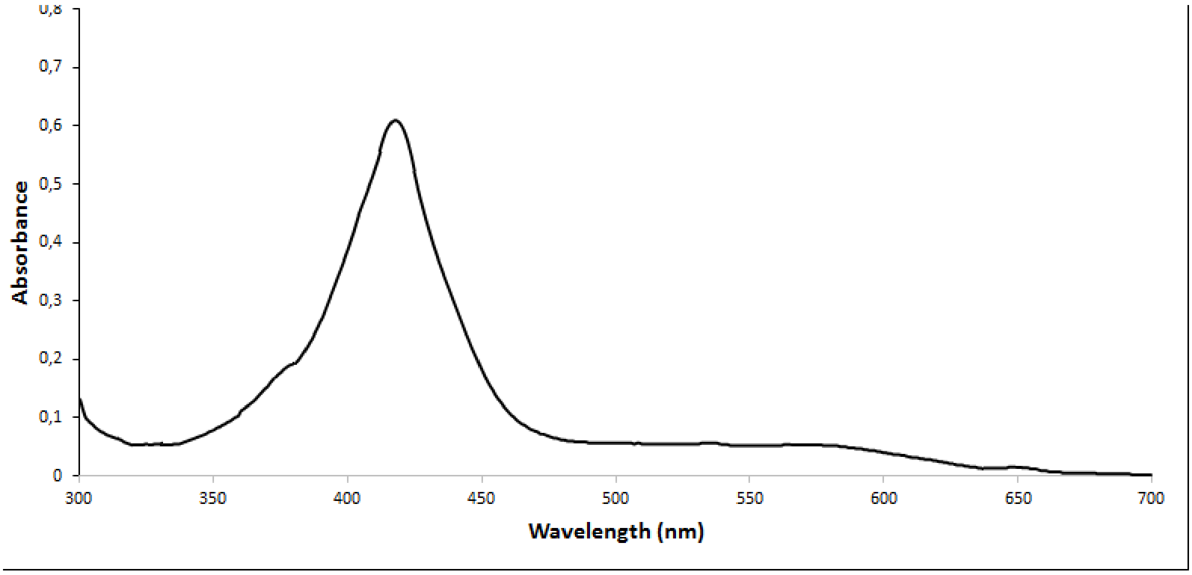

Figure S17: Electronic absorption spectra of **2d** in $CHCl_3$.

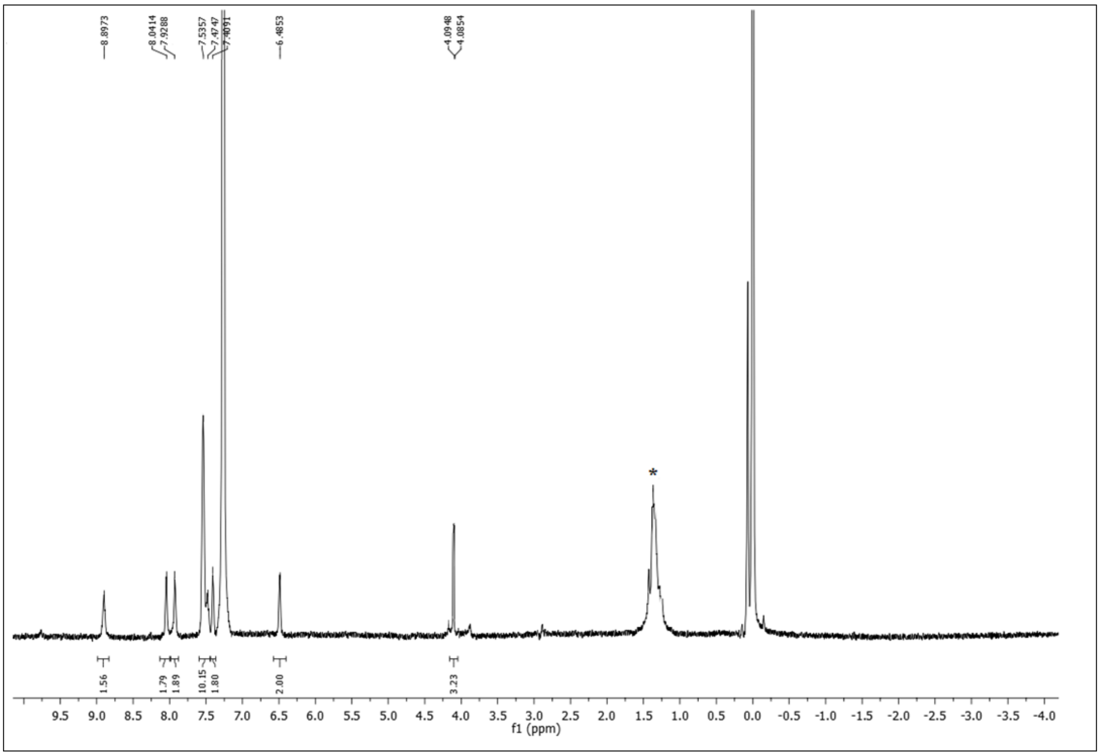

Figure S18: ^{1}H NMR spectrum of **2e** (−4–10 ppm, Solvent: $CDCl_3$).

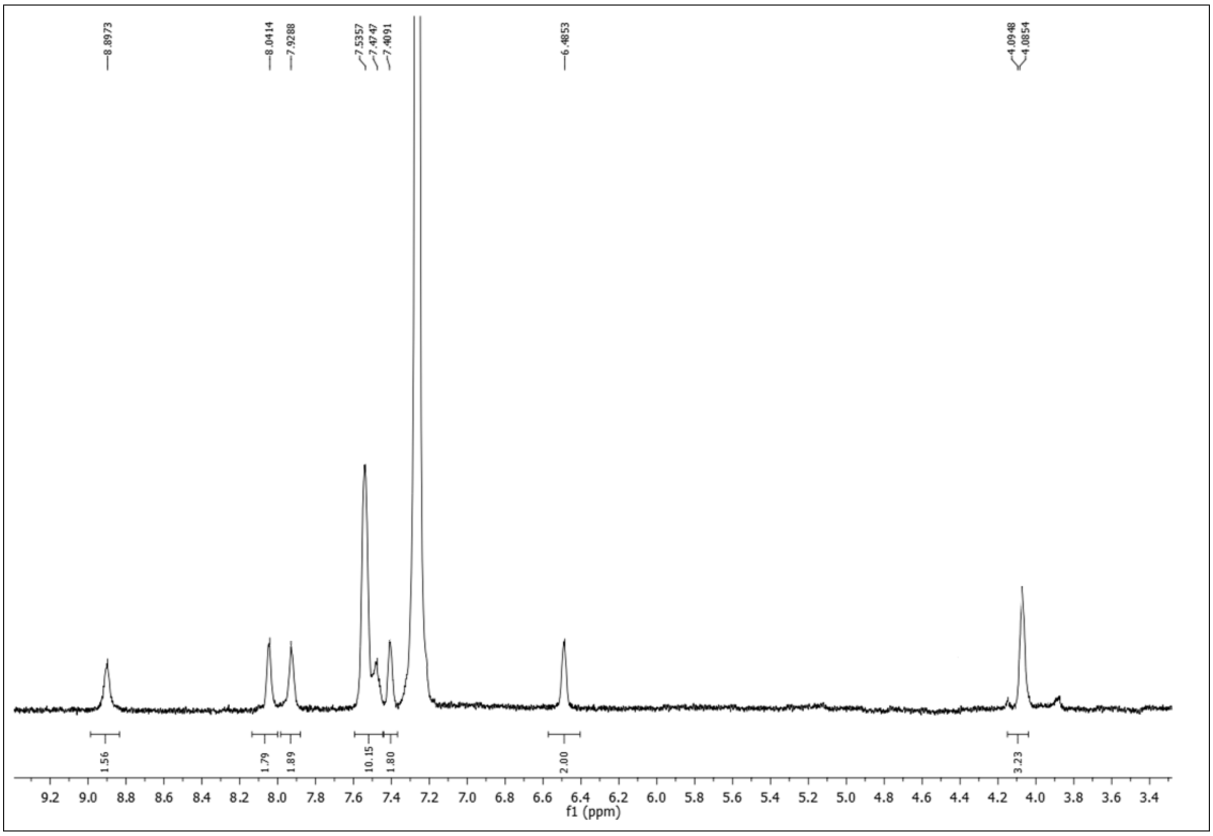

Figure S19: ^{1}H NMR spectrum of **2e** (3.3–9.3 ppm, Solvent: $CDCl_3$).

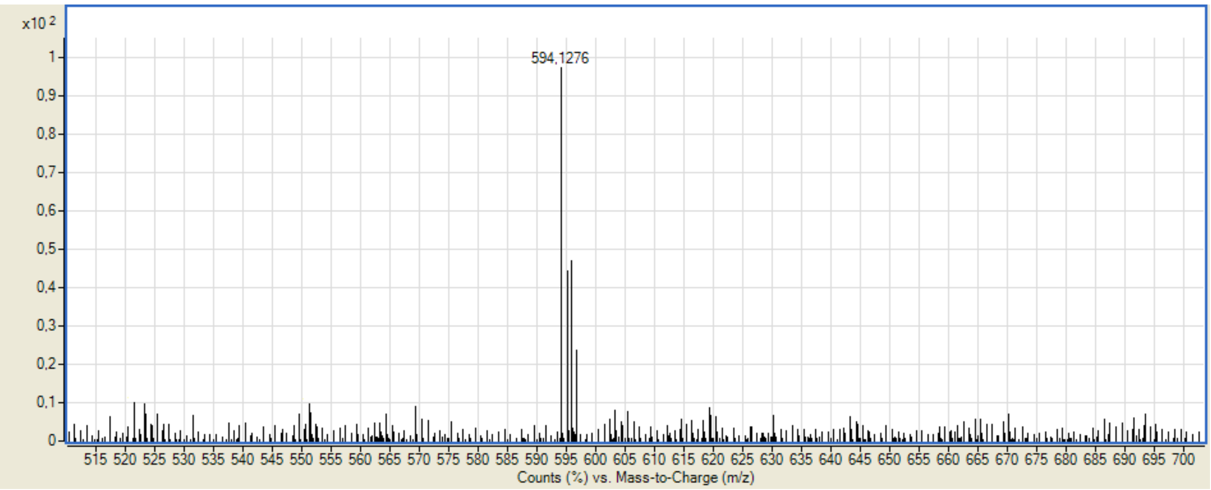

Figure S20: ESI HRMS (positive mode) of compound **2e**.

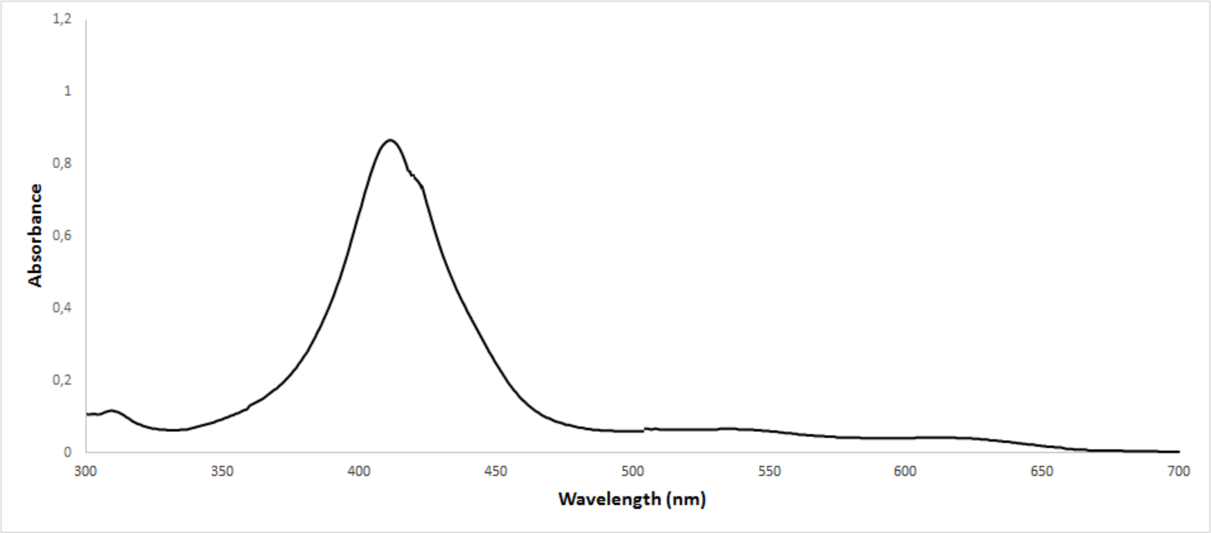

Figure S21: Electronic absorption spectra of **2e** in $CHCl_3$.

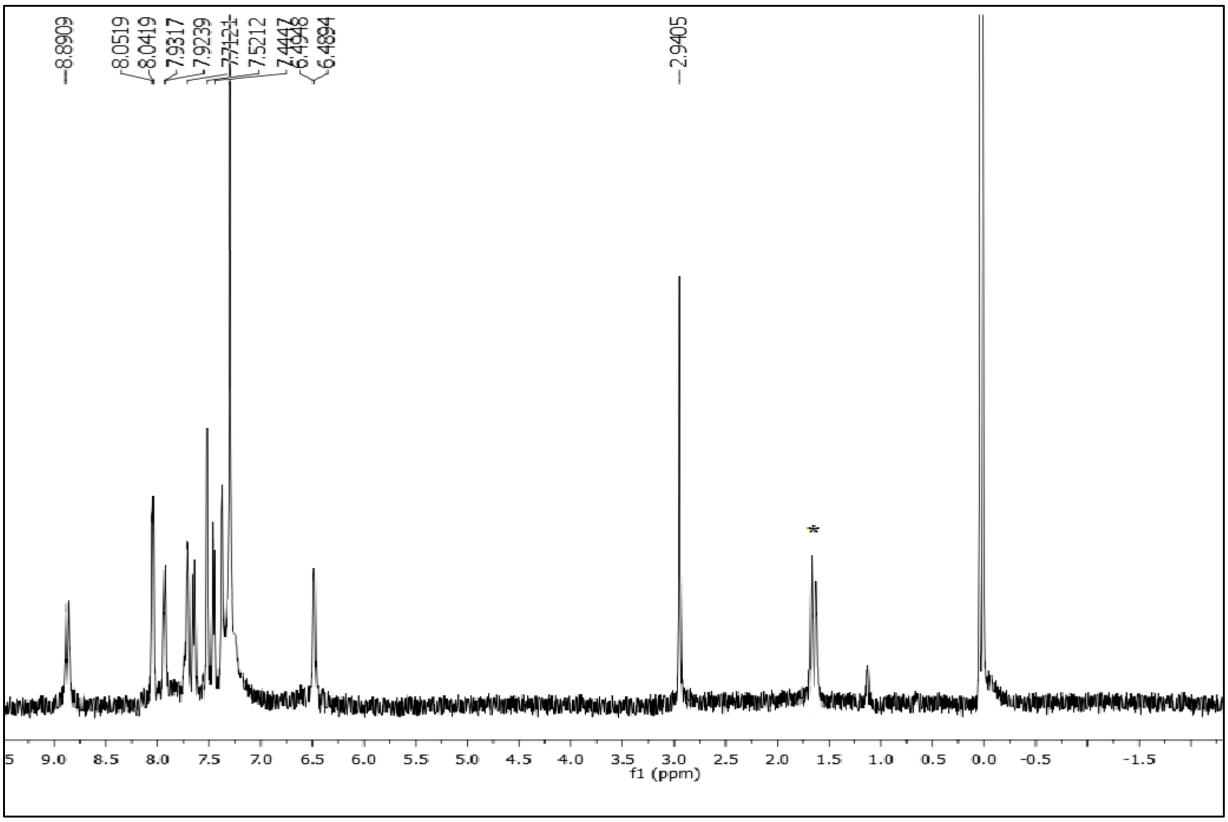

Figure S22: ^{1}H NMR spectrum of **2f** (−2–10 ppm, Solvent: $CDCl_3$).

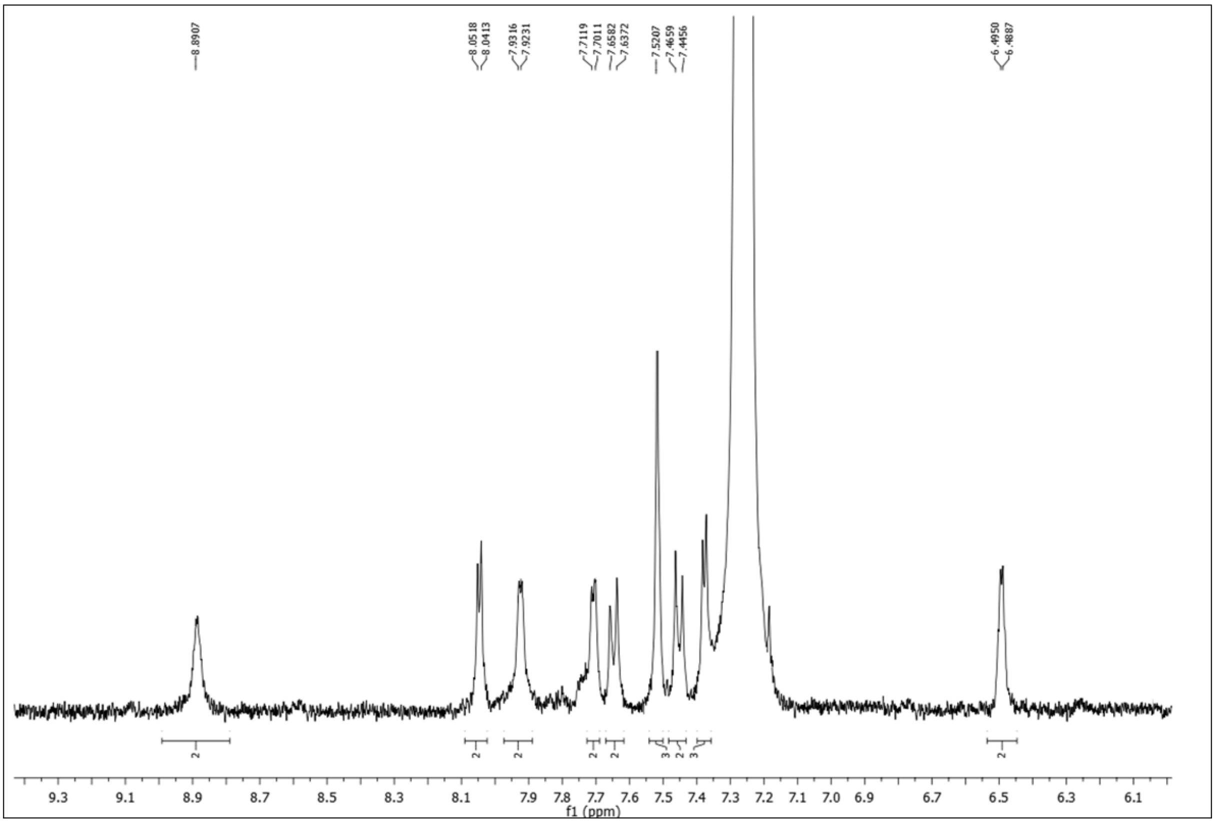

Figure S23: ^{1}H NMR spectrum of **2f** (6.0–9.3 ppm, Solvent: $CDCl_3$).

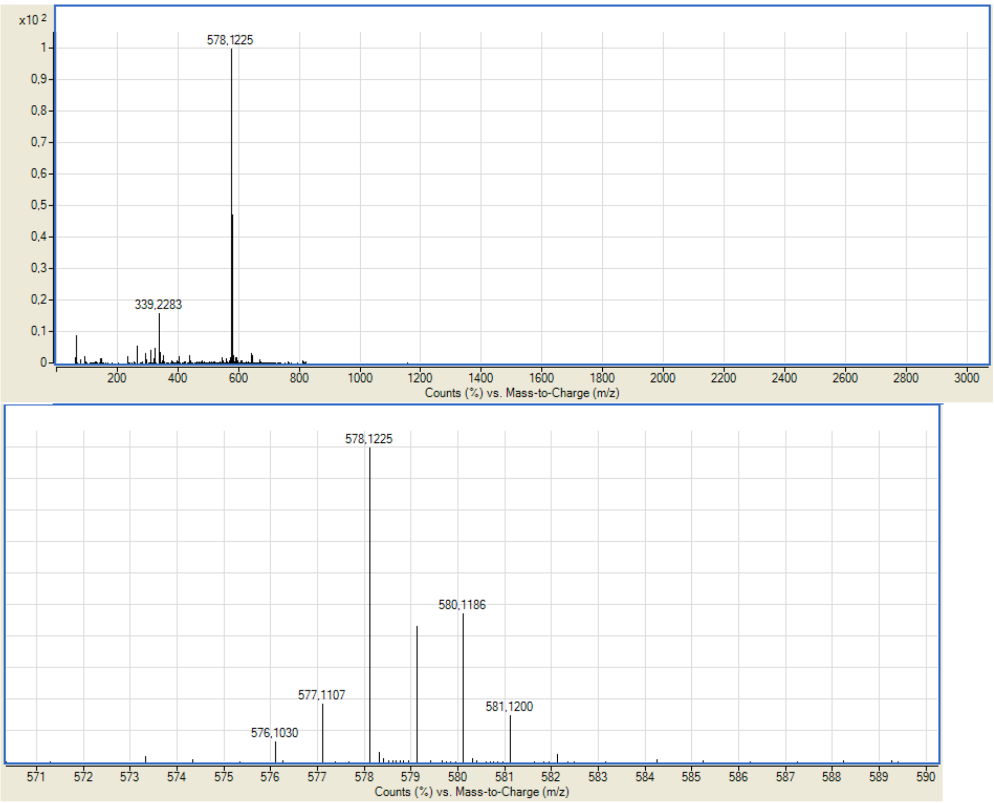

Figure S24: ESI HRMS (positive mode) of compound **2f**.

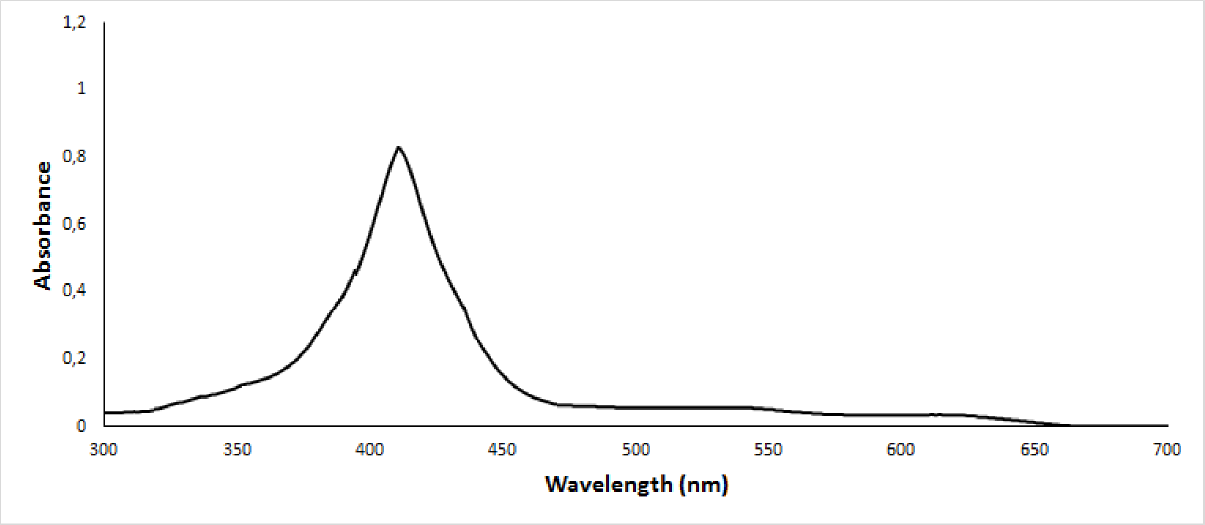

Figure S25: Electronic absorption spectra of **2f** in $CHCl_3$.

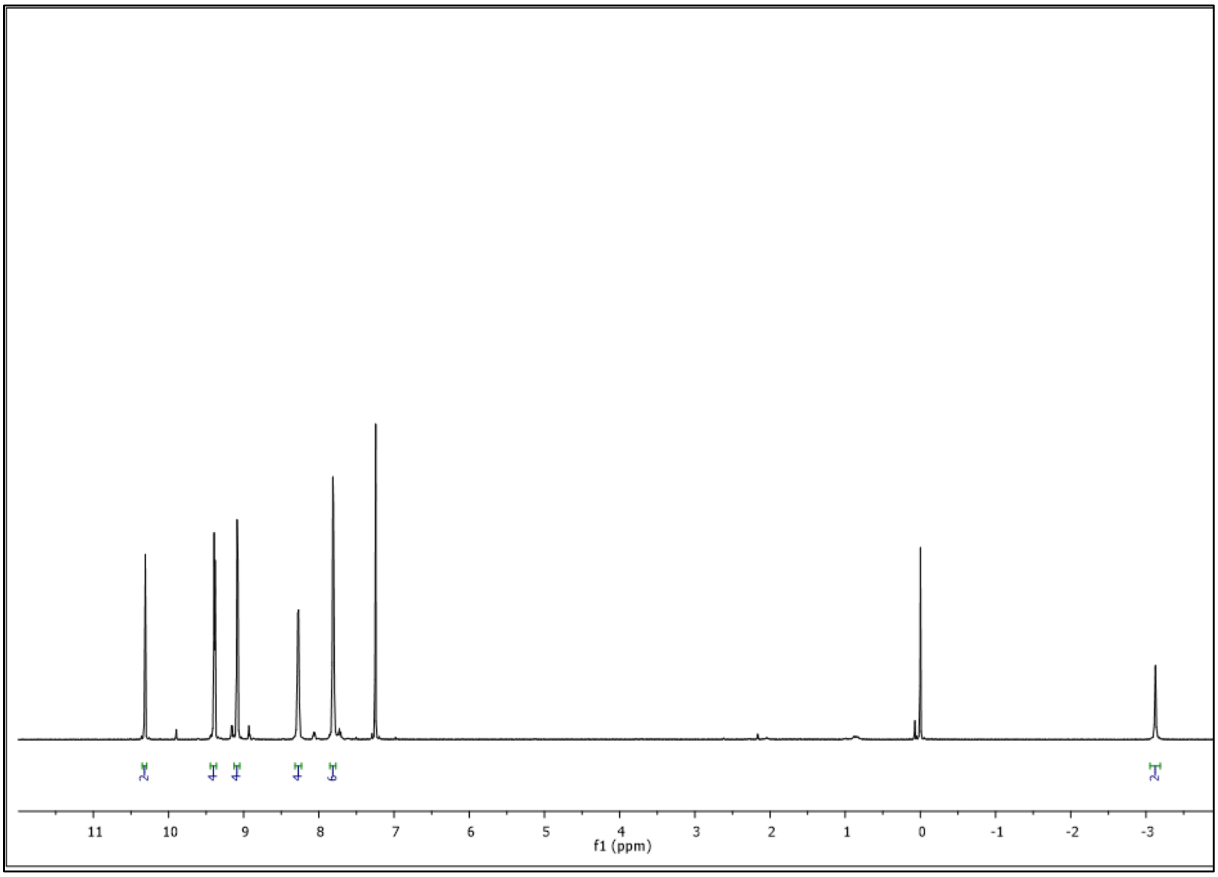

Figure S26: ^{1}H NMR spectrum of **3** (Solvent: $CDCl_3$).

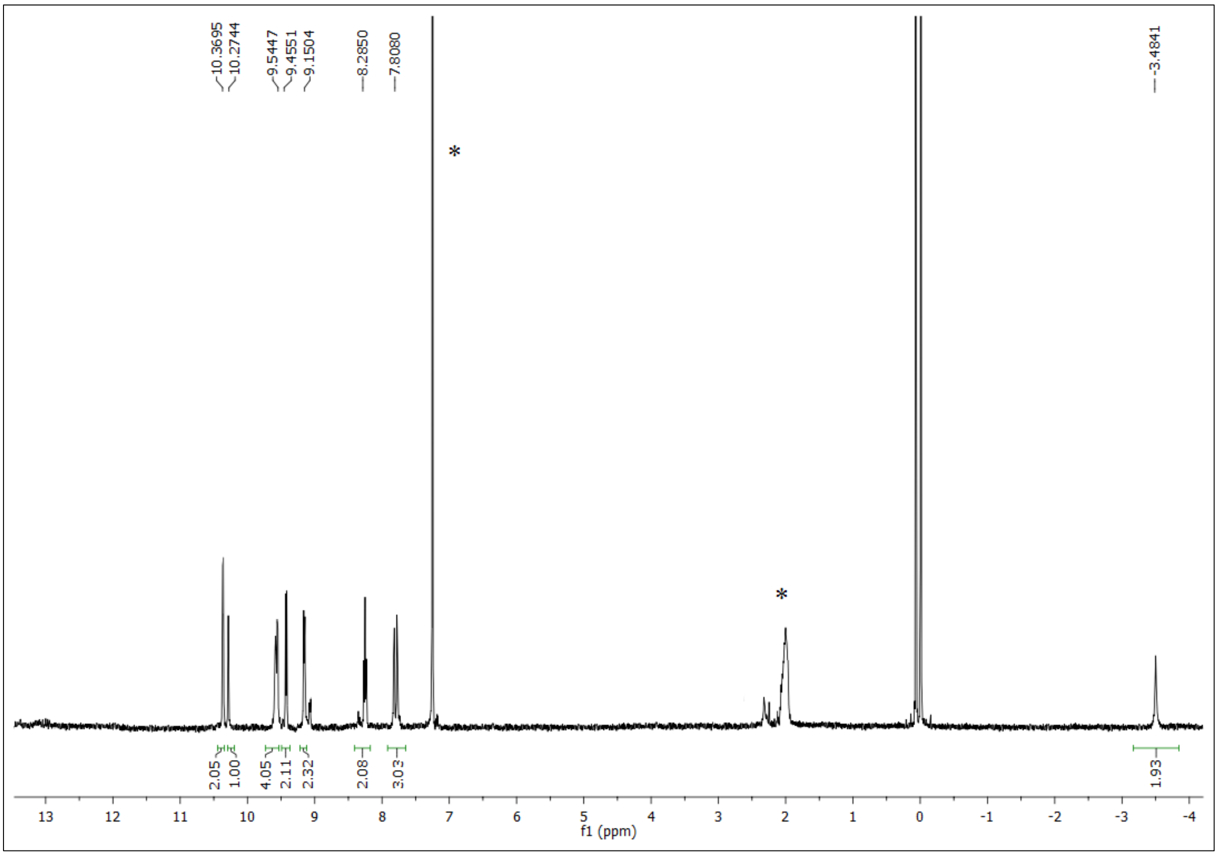

Figure S27: ^{1}H NMR spectrum of **4** (Solvent: $CDCl_3$).

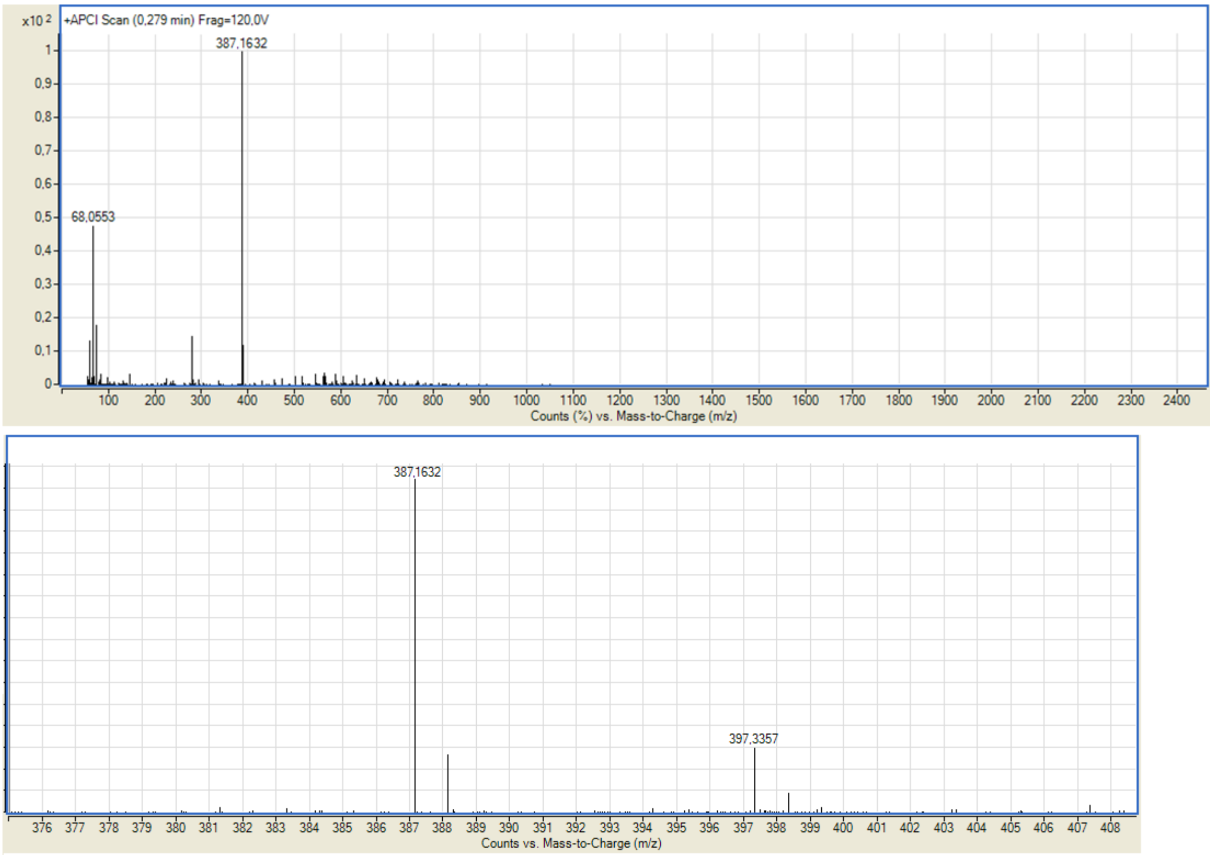

Figure S28: ESI HRMS (positive mode) of compound **4**.

References

1. Brückner, C.; Posakony, J. J.; Johnson, C. K.; Boyle, R. W.; James, B. R.; Dolphin, D. *J. Porphyrins Phthalocyanines* **1998**, *2*, 455-465.

2. Ryppa, C.; Senge, M. O.; Hatscher, S. S.; Kleinpeter, E.; Wacker, P.; Schilde, U.; Wiehe, A. *Chem. Eur. J.* **2005**, *11*, 3427-3442.
